# Investigating thermal properties of 2D non-layered material using a NEMS-based 2-DOF approach towards ultrahigh-performance bolometer

**DOI:** 10.1093/nsr/nwae248

**Published:** 2024-07-17

**Authors:** Luming Wang, Song Wu, Zejuan Zhang, Jiankai Zhu, Luwei Zou, Bo Xu, Jiaqi Wu, Junzhi Zhu, Fei Xiao, Chenyin Jiao, Shenghai Pei, Jiaze Qin, Yu Zhou, Juan Xia, Zenghui Wang

**Affiliations:** Institute of Fundamental and Frontier Sciences, University of Electronic Science and Technology of China, Chengdu 610054, China; Institute of Fundamental and Frontier Sciences, University of Electronic Science and Technology of China, Chengdu 610054, China; Institute of Fundamental and Frontier Sciences, University of Electronic Science and Technology of China, Chengdu 610054, China; Institute of Fundamental and Frontier Sciences, University of Electronic Science and Technology of China, Chengdu 610054, China; School of Physics, Hunan Key Laboratory of Nanophotonics and Devices, Central South University, Changsha 410083, China; Institute of Fundamental and Frontier Sciences, University of Electronic Science and Technology of China, Chengdu 610054, China; Institute of Fundamental and Frontier Sciences, University of Electronic Science and Technology of China, Chengdu 610054, China; Institute of Fundamental and Frontier Sciences, University of Electronic Science and Technology of China, Chengdu 610054, China; Institute of Fundamental and Frontier Sciences, University of Electronic Science and Technology of China, Chengdu 610054, China; Institute of Fundamental and Frontier Sciences, University of Electronic Science and Technology of China, Chengdu 610054, China; Institute of Fundamental and Frontier Sciences, University of Electronic Science and Technology of China, Chengdu 610054, China; Institute of Fundamental and Frontier Sciences, University of Electronic Science and Technology of China, Chengdu 610054, China; School of Physics, Hunan Key Laboratory of Nanophotonics and Devices, Central South University, Changsha 410083, China; Institute of Fundamental and Frontier Sciences, University of Electronic Science and Technology of China, Chengdu 610054, China; Institute of Fundamental and Frontier Sciences, University of Electronic Science and Technology of China, Chengdu 610054, China; State Key Laboratory of Electronic Thin Films and Integrated Devices, University of Electronic Science and Technology of China, Chengdu 611731, China

**Keywords:** thermal properties, non-layered material, resonant NEMS, nanoscale motion, bolometer

## Abstract

Two-dimensional (2D) non-layered materials in many aspects differ from their layered counterparts, and the exploration of their physical properties has produced many intriguing findings. However, due to challenges in applying existing experimental techniques to such nanoscale samples, their thermal properties have remained largely uncharacterized, hindering further exploration and device application using this promising material system. Here, we demonstrate an experimental study of thermal conduction in *β*-In_2_S_3_, a typical non-layered 2D material, using a resonant nanoelectromechanical systems (NEMS) platform. We devise a new two-degrees-of-freedom technique, more responsive and sensitive than Raman spectroscopy, to simultaneously determine both the thermal conductivity to be 3.7 W m^−1^ K^−1^ and its interfacial thermal conductance with SiO_2_ as 6.4 MW m^−2^ K^−1^. Leveraging such unique thermal properties, we further demonstrate a record-high power-to-frequency responsivity of −447 ppm/μW in *β*-In_2_S_3_ NEMS sensors, the best among drumhead NEMS-based bolometers. Our findings offer an effective approach for studying thermal properties and exploring potential thermal applications of 2D non-layered materials.

## Teaser

This work employs a NEMS platform to probe the thermal conduction at the nanoscale, offering a new approach for extracting thermal properties in ultrathin crystals.

## INTRODUCTION

Thermal conduction plays a critical role in the performance of semiconductor devices, as noise figure, power consumption and reliability of devices are all sensitive to temperature and thus thermal conduction [1–3]. The introduction of two-dimensional (2D) semiconductors [4] and their heterostructures [5] offers new opportunities for designing nanoscale devices with unique performance, accompanied by the average phonon mean-free path exceeding or equaling device thickness, making collective phonon excitations the main heat carriers and thermal conductivity tunable by device size [6–8]. This also introduces a plethora of intriguing thermal behavior in 2D devices, including enhanced phonon-boundary scattering at the atomically sharp interfaces between the 2D materials and their immediate environment [9,10], alterations in thermal scattering rates due to changes in crystal symmetry [11], localized temperature rise effects (hotspots) in electronic and optoelectronic devices [12], and huge thermal tunability in the nanoelectromechanical systems (NEMS) [13–15]. Consequently, investigation of thermal conduction in 2D materials is crucial for ensuring the performance of 2D semiconductor devices and achieving optimal thermal management of these devices.

In contrast to 2D layered materials, which are stacked through van der Waals interactions, 2D non-layered materials are unique in that they are formed by chemical bonds in all three dimensions. This is expected to give rise to intriguing properties in thermal conduction, thermal stability and phonon dissipation. Further, as the thickness decreases to nanoscale, 2D non-layered materials undergo bond breakage, crystal rearrangement and structural deformation [16], resulting in excellent tunability in their band structures, electrical conductivity and ferromagnetism [17–20]. Moreover, the abundance of surface dangling bonds facilitates unique surface activity on these materials, rendering them highly promising for sensing applications and potentially useful in heterogeneous integration [21–24], all of which are affected by and can be further combined with their unique thermal properties to enable new device designs. Therefore, understanding the thermal conduction process in 2D non-layered materials is essential for the full exploitation of the aforementioned outstanding properties and for enabling new applications.

To date, however, the research on thermal conduction in 2D non-layered materials is largely absent, and the utilization of their thermal capabilities remains to be explored. The challenge in experimental techniques is a key hindering factor. Due to the minuscule sample sizes, especially the atomic-scale thinness, conventional thermal conductivity measurement techniques, such as laser flash and 3*ω* methods, are not suitable for measuring in-plane thermal conductivity in these nanoscale samples [25,26]. While some alternatives, such as thermal bridge, Raman spectroscopy and scanning thermal microscopy (SThM), have been developed for measuring thermal parameters in layered 2D materials [8,27–31], such methods can only determine one unknown thermal parameter at a time, often requiring pre-knowledge of another thermal conduction coefficient, such as one derived from theory. Therefore, the recent emergence of 2D non-layered materials has faced greater challenges with regard to studying their thermal properties, largely due to the simultaneous absence of any knowledge about their thermal conductivity and their interfacial thermal conductance with heat source/substrate. This impedes the distinction between their effects in thermal measurements, further hindering the decoupling and quantification of the different thermal conduction coefficients. In addition, the responsivity (output-to-input ratio, i.e. gain) and sensitivity (smallest measurable signal) of many existing thermal-transport techniques in nanoscale samples have also hampered the accurate extraction of thermal parameters.

In this work, we study thermal conduction of *β*-In_2_S_3_ (a typical non-layered 2D material) using a resonant NEMS platform with a variable laser heat source. We leverage two degrees of freedom (DOF) in the 2D NEMS platform, laser position and laser power, to simultaneously determine both the thermal conductivity *κ* of *β*-In_2_S_3_ as 3.7 W m^−1^ K^−1^ and the interfacial thermal conductance *G*_B_ between *β*-In_2_S_3_ and SiO_2_ as 6.4 MW m^−2^ K^−1^. For this specific type of non-layered 2D material, such a NEMS-based 2-DOF approach is estimated to be 1 million times more responsive and >2000 times more sensitive than the Raman-spectroscopy-based method. Furthermore, we demonstrate that the unusual thermal conductivity translates to an outstanding power-to-frequency responsivity of the *β*-In_2_S_3_ resonator, reaching −447 ppm/μW at 532 nm, the best among drumhead NEMS-based bolometers. Our findings provide insights into the thermal properties of 2D *β*-In_2_S_3_ and offer opportunities for exploring potential thermal applications for 2D non-layered materials.

## RESULTS AND DISCUSSION

**Figure 1.**
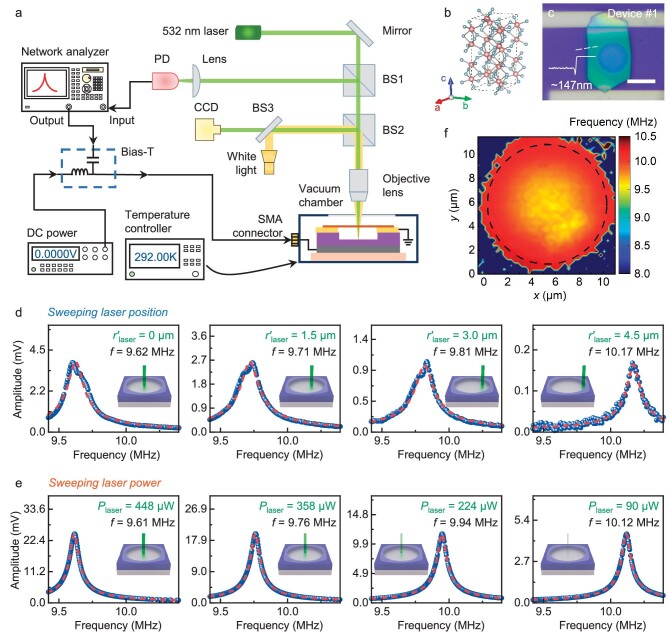
Resonance measurement setup and resonance response with varying laser position/laser power. (a) Schematic of the custom-built resonant NEMS measurement system [39,40]. PD: photodetector, BS: beam splitter. (b) Illustration of a unit cell in the *β*-In_2_S_3_ lattice, with larger and smaller spheres symbolizing In and S atoms, respectively. (c) An optical image of a representative *β*-In_2_S_3_ resonator with a diameter of 10 μm and a thickness of ∼147 nm. Scale bar: 10 μm. (d–e) Measured resonance response (blue spheres) with (d) various laser positions (along the radius) and (e) various laser powers. The dashed curves (red) represent the fitting results. (f) Frequency mapping of Device #1. The mapping region spans 11.5 μm × 11.5 μm, and the dashed outline delineates the suspended area (see Section S9 for details).

We use a custom-built 2D NEMS platform to explore the thermal response of 2D devices using resonance frequency shifts. As depicted in Fig. 1a, a 532 nm power-tunable laser, with a spot size of ∼1.4 μm (Methods), serves two functions at the same time, as a detection laser for measuring resonance motion [32–34] (through laser interferometry [35–37], see Section S1), and as a source of thermal flux (through optothermal effect, see Sections S2 and S3). All devices are measured under vacuum (∼1 × 10^−5^ Torr), and a displacement stage with a 0.3 μm resolution is employed to precisely control the device position. The devices we use are *β*-In_2_S_3_ (a prototypical non-layered 2D semiconductor) drumhead resonators. Figure 1b and c show the crystal structure and optical image of a representative device (Device #1, ∼147 nm thick and 10 μm in diameter). It is important to note that both the thermal conductivity *κ* of 2D *β*-In_2_S_3_ and the interfacial thermal conductance *G*_B_ between 2D *β*-In_2_S_3_ and SiO_2_ have not yet been reported, which prevents the application of established measurement techniques.

To address this challenge, we exploit two DOFs enabled by the 2D NEMS platform in order to simultaneously extract the values of *κ* and *G*_B_. Specifically, we vary both laser position and laser power when measuring the fundamental mode resonance frequency *f*_0_ for the devices, and the measurement results for Device #1 are shown in Fig. 1d−f. We make two observations from the data: first, the device frequency increases as the laser spot moves from device center towards the edge; second, the frequency increases as the laser power decreases. The high responsivity of resonance frequency to both the DOFs suggests that *f*_0_ is an effective indicator for exploring thermal properties. Furthermore, we conduct a mapping of resonance frequency *f*_0_ while maintaining a fixed laser power (448 μW), with results shown in Fig. 1f. We find that the *f*_0_ map is azimuthally symmetric, with a minimum *f*_0_ at the center. This observation confirms that the in-plane thermal property of 2D *β*-In_2_S_3_ crystal is isotropic (otherwise the mapping result would not exhibit a circularly symmetric pattern [38]), thus we can use just one set of *κ* and *G*_B_ values to describe the system.

**Figure 2.**
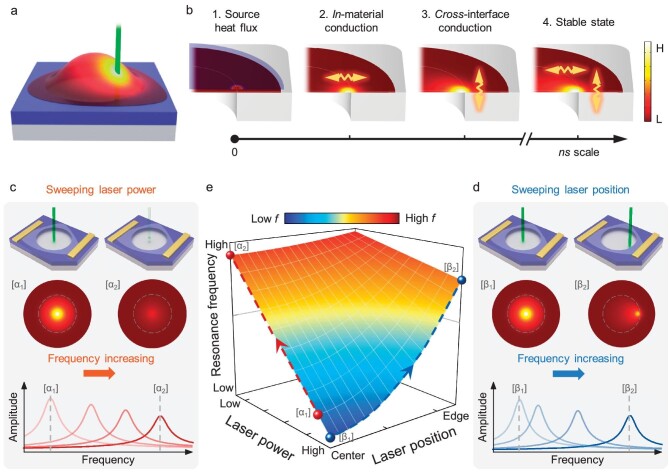
Mechanism of laser-heating-induced frequency tuning. (a) Illustration of a fully clamped 2D-material-based resonator vibrating in fundamental mode. Laser heating (green vertical beam) generates a temperature gradient across the membrane (depicted by the yellow-red colour). (b) Modeling of the in-device thermal conduction process. Upon laser illumination (scene 1), heat initially conducts across the in-plane direction of the drumhead, dominated by the thermal conductivity *κ* (scene 2). Subsequently, heat conducts across the supported region and establishes a thermal pathway to the underlying substrate (SiO_2_) through the interfacial thermal conductance *G*_B_ (scene 3). The device reaches a thermally steady state in a matter of nanoseconds (scene 4). (c–d) Theoretical analyses of the frequency tuning process while (c) sweeping the laser power and (d) sweeping the laser position. The color scale of the temperature distribution maps is consistent with that in (b). (e) 3D plot by amalgamating both mechanisms. When sweeping the laser position, as depicted in (d), the resonance frequency shifts as the laser moves from the center [*β*_1_] to the edge [*β*_2_] along the blue dashed line. A similar trajectory, as illustrated in (c), is presented with the red spheres ([*α*_1_] and [*α*_2_]) and the red dashed line.

In order to quantify the relationship between thermal conduction and resonance frequency, we first analyze the optothermal process (Fig. 2a). As shown in Fig. 2b, once the laser is incident onto the device (scene 1), heat conduction initially occurs (scene 2) within the 2D material (primarily governed by its in-plane thermal conductivity), and then between the 2D material and the substrate, with the interfacial thermal conductance becoming important (scene 3). Within nanoseconds, thermal equilibrium is established (scene 4), resulting in a stable temperature distribution with an average temperature increase Δ*T*_avg_.

For a drumhead resonator with a thickness of *t* and an area of *A*, the optothermal surface tension *γ*_th_ (in N/m, also averaged over the entire device) induced by Δ*T*_avg_ is [38,41]:


(1) \begin{eqnarray*}
{{\gamma }_{{\mathrm{th}}}} = \frac{t}{A}\int\!\!\!\!\int_A {\frac{2}{3}}\sigma {\mathrm{d}}x{\mathrm{d}}y = - \frac{{2t{{E}_{\mathrm{Y}}}\alpha \Delta {{T}_{{\mathrm{avg}}}}}}{{3\left( {1 - v} \right)}},
\end{eqnarray*}

where *σ* is the thermally induced stress per unit length (in N/m), *α* is the thermal expansion coefficient of the 2D material, and *E*_Y_ and *v* are the Young's modulus and Poisson's ratio of the material, respectively. We use *α* = 10 ppm/K and *E*_Y_ = 60 GPa for a 2D *β*-In_2_S_3_ crystal, both derived from our measurements (see Sections S4 and S5). The minus sign indicates that a larger Δ*T*_avg_ leads to a more negative *γ*_th_ (less total tension).

To simplify the analysis, we follow the convention of assuming that *E*_Y_ remains mostly constant with temperature [42,43]. Taking into account the influence of the thermal stress *γ*_th_, the resonance frequency (see Section S6) becomes [44–46]:


(2) \begin{eqnarray*}
{{f}_0}\!=\! \left(\frac{{kd}}{{4\pi }}\right)\sqrt {\frac{{{\mathrm{16}}D}}{{{{\rho }_{2D}}{{d}^{\mathrm{4}}}}}\left[\left(\frac{{kd}}{{\mathrm{2}}}\right)^{\mathrm{2}}\! +\!\frac{{\left( {{{\gamma }_0} + {{\gamma }_{{\mathrm{th}}}}} \right){{d}^{\mathrm{2}}}}}{{{\mathrm{4}}D}}\right]},
\end{eqnarray*}

where *k* is a modal parameter determined numerically [47,48], and *γ*_0_ is the initial surface tension (assumed to be uniform across the device) without thermal stress (in N/m). The flexural rigidity *D* is given by *D* = *E*_Y_*t*^3^/[12(1–*ν*^2^)], and *ρ*_2D_ denotes the areal mass density (for *β*-In_2_S_3_, *ρ*_3D_ = *ρ*_2D_/*t* = 4.613 g/cm^3^) [49]. Since *γ*_th_ is negative for positive Δ*T*_avg_, laser heating always leads to decreased *f*_0_.

From the above analysis (see Section S6 for detailed calculations), we can explain the experimental observations (Fig. 1d−f): when the laser power is decreased, it reduces Δ*T*_avg_ and thus increases total tension *γ*_0_ + *γ*_th_, causing *f*_0_ to increase (Fig. 2c). Similarly, when the laser spot moves toward the device edge, the heat dissipation into the substrate becomes easier, which reduces Δ*T*_avg_ and increases *γ*_0_ + *γ*_th_, also leading to a higher *f*_0_ (Fig. 2d). To better illustrate such an optothermal response in *f*_0_, we construct a 3D plot (Fig. 2e) to visualize the entire 2-DOF parameter space, using the above equations. Each set of measurements (varying laser position or power) corresponds to a vertical slice on this curved surface, as indicated by the curved lines of different orientations on this curved surface.

**Figure 3.**
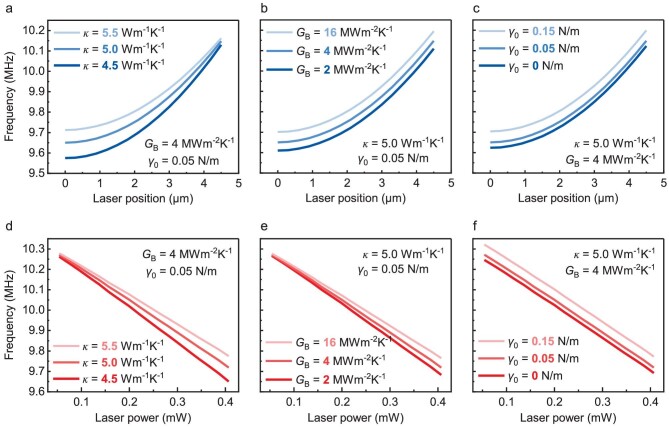
Exploring the effects of *κ, G*_B_ and *γ*_0_ on position and power response curves. (a−c) Theoretical frequency responses to laser position for various (a) thermal conductivities *κ*, (b) interfacial thermal conductance *G*_B_, and (c) initial in-plane stress *γ*_0_. (d−f) Theoretical frequency responses to laser power for various (d) thermal conductivities *κ*, (e) interfacial thermal conductance *G*_B_, and (f) initial in-plane stress *γ*_0_. Parameters of Device #1 are used in simulations.

In order to extract the thermal conductivity *κ* of *β*-In_2_S_3_ and the interfacial thermal conductance *G*_B_ between *β*-In_2_S_3_ and SiO_2_, we examine how this curved surface evolves in response to *κ* and *G*_B_. To quantify the contribution from individual parameters, we focus on those vertical slices (along both directions) of such 3D stack to decouple the effects from the two experimental DOFs, laser position and power (curves in Fig. 3). This will also allow direct comparison to the experimental results.

We first direct our attention to the ‘position’ curves (Fig. 3a−c). We observe that *κ* plays a unique role in this case: it can affect the curvature and steepness of the curve, whereas *G*_B_ and *γ*_0_ mostly just translate the curve. This suggests that by fitting measurements to the calculated ‘position’ curve, we can precisely extract the value of *κ* even with just rough ranges for *G*_B_ and *γ*_0_. Next, by examining the ‘power’ curves (Fig. 3d−f), we note that they are almost linear (negligible curvature), with their slopes tuned by *κ* and *G*_B_, but not *γ*_0_. Consequently, upon determining the value of *κ*, we can further extract *G*_B_ based on the slope of the ‘power’ curve, independent of device initial tension *γ*_0_. Therefore, with such 2-DOF measurements and analysis, one can simultaneously extract both of the unknown thermal parameters (see Section S7 for detailed discussion). This also offers the advantage that even if one of the parameters such as the thermal contact resistance is affected by clamping conditions, the other thermal parameter such as thermal conductivity can still be independently and reliably extracted.

**Figure 4.**
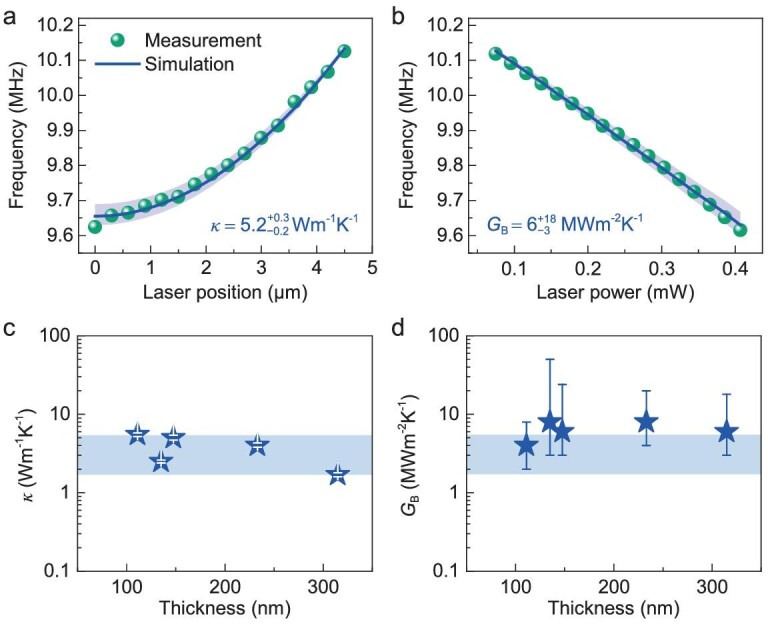
Extraction of thermal conductivity *κ* and thermal conductance *G*_B_. (a, b) Measured (green spheres) and simulated (blue lines) frequency response of Device #1 vs. laser position and laser power. The best-fit simulation yields a *k* value of 5.2 W m^−1^ K^−1^ and a *G*_B_ value of 6 MW m^−2^ K^−1^. (c, d) Summary for *κ* and *G*_B_ values extracted from all devices (see Table S2 for a complete list).

We now compare the measurement results (spheres in Fig. 4a and b) to the calculated results (lines). First, by fitting data to the ‘position’ curve (Fig. 4a) we are able to directly extract the in-plane thermal conductivity of *β*-In_2_S_3_, *κ* = 5.2 W m^−1^ K^−1^, with a fitting that results in an *R*^2^ = 0.9946 (see Section S8 for details and estimation of uncertainties). Next, by performing data analysis using the ‘power’ curve, we obtain the interfacial thermal conductance between *β*-In_2_S_3_ and SiO_2_ as *G*_B_ = −6 MW m^−2^ K^−1^ with an *R*^2^ = 0.9981. This clearly demonstrates the effectiveness of our 2-DOF method using such a NEMS platform. The extracted thermal transport properties, which do not exhibit clear dependence on thickness for samples up to a few hundred nanometers, are consistent with expectations from molecular dynamic (MD) studies and other experimental investigations in the literature [8,50,51,52]. We further apply this method to multiple 2D *β*-In_2_S_3_ resonators of different sizes (see Section S9 and Table S2 for detailed device information), and we find that the extracted thermal conductivity values are reasonably close to each other with a *κ*_avg_ = 3.7 W m^−1^ K^−1^, and an interfacial thermal conductance *G*_B,avg_ = 6.4 MW m^−2^ K^−1^ (Fig. 4c and d).

**Figure 5.**
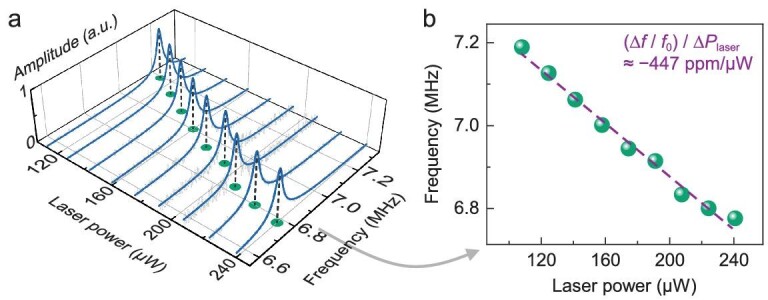
Demonstration of a high-performance bolometer. (a) Evolution of normalized resonance response with increasing laser power. The blue solid lines represent the fitting results using the simple harmonic oscillator model, while the measured data are shown in light gray lines. (b) The resonance frequency decreases with increasing laser power, suggesting excellent bolometric performance with a power-to-frequency responsivity of –447 ppm/μW. The green spheres are the frequencies derived from (a), and the purple dashed line shows the linear fit.

It is worth noting that the unusual thermal conductivity of 2D *β*-In_2_S_3_, significantly lower than that of layered 2D materials [27–29,53,54], makes such NEMS devices much more prone to optothermal frequency shifts. This can translate to high power-to-frequency responsivity in NEMS optical sensors. Here we show the data from device #5 (Fig. 5) functioning as a bolometer, which exhibits a significant response from 6.78 MHz to 7.19 MHz over a laser power variation of just 0.133 mW. This constitutes a record-high responsivity of –447 ppm/μW at room temperature, the best reported to date among all drumhead NEMS-based light power meters [15,55–58] (see Section S10 and S11). This demonstrates the great promise of 2D non-layered devices in enabling high-performance light sensors.

The above results, in turn, further highlight some of the key advantages of our approach, i.e. superb responsivity and sensitivity, which in principle can be applied to different types of 2D crystals. Critical to any measurement technique is the detection efficiency (responsivity) and limit (sensitivity). Here we compare our technique with the Raman spectroscopy method previously reported for studying thermal properties in 2D layered materials (see Section S12 for details). We measure the maximum power-to-frequency responsivity of the method, which relies on detecting Raman peak shift upon heating, to be 0.0047 ppm/μW. This demonstrates that our NEMS approach is 1 million times more responsive for a given change in laser power. Furthermore, we estimate the measurement sensitivity (minimum resolvable laser power change, see Section S13 for details) of the two methods, and find that our approach is >2000 times more sensitive than the spectroscopy method (0.11 μW vs. 242 μW). This again demonstrates the excellent responsivity and sensitivity of the NEMS technique, which allows efficient and reliable (see Section S14 for details) extraction of the unknown thermal properties in emerging nanomaterials.

In summary, we experimentally determine the thermal properties in 2D non-layered material, *β*-In_2_S_3_. We demonstrate an effective NEMS-based method, which leverages two DOFs in the measurement (laser position and laser power) to simultaneously determine multiple unknown thermal parameters, and is 1 million times more responsive and >2000 times more sensitive than Raman spectroscopy. Based on this method, we extract the thermal conductivity of 2D *β*-In_2_S_3_ crystal as *κ*_avg_ = 3.7 W m^−1^ K^−1^, and the interfacial thermal conductance between *β*-In_2_S_3_ and SiO_2_ as *G*_B,avg_ = 6.4 MW m^−2^ K^−1^. Interestingly and importantly, the low thermal conductivity of 2D *β*-In_2_S_3_ translates to an excellent power-to-frequency responsivity of –447 ppm/μW, far surpassing the performance of all other drumhead NEMS-based bolometers. In addition, our findings could inspire further exploration on thermal properties of both layered and non-layered 2D materials, which could lead to an improved understanding of the underlying physics, such as the roles of electrons and phonons in the thermal transport of atomically thin crystals. Our work can offer important guidelines for studying thermal properties in nanoscale samples using the NEMS platform, and provide valuable insights into the design and development of 2D non-layered sensing and signal processing devices [59].

## METHODS

### Sample preparation

We synthesize *β*-In_2_S_3_ nanoflakes using a controlled heating system equipped with a 21-mm-diameter quartz tube under ambient pressure. The indium trifluoride (InF_3_) and sulfur (S) powders, serving as precursors, are placed in the center of the heating furnace and out of the central hot zone, respectively. A fluorophlogopite mica [KMg_3_AlSi_3_O_10_F_2_] substrate with a size of 10 mm × 10 mm is placed at the center of the heating furnace above the InF_3_ powder. Before heating, the whole system is purged with 200 sccm Ar for 10 minutes. Then the furnace is heated to 750^º^C at a rate of 40^º^C/min with Ar flow of 40 sccm, and maintained for 5 minutes for the growth of *β*-In_2_S_3_. The temperature of the sulfur is maintained at 180–200^º^C through the growth stage. After growth, the heating belt for the sulfur is shut down immediately and the furnace is cooled to room temperature.

### Device fabrication

We employ a water-assisted lift-off and dry transfer technique to fabricate *β*-In_2_S_3_ NEMS resonators. Instead of cleaving bulk crystals, we start with synthetic *β*-In_2_S_3_ nanoflakes [60] and transfer them onto a polydimethylsiloxane (PDMS) stamp using water-assisted lift-off. We then select uniform large-sized *β*-In_2_S_3_ flakes and transfer each flake to a pre-patterned circular microtrench on a SiO_2_/Si substrate. In this process, we adjust the alignment of the flakes to ensure their contact with the pre-fabricated metal electrodes, enabling electrical excitation of the devices.

### Raman measurements

The *β*-In_2_S_3_ sample is measured using a continuous wave laser at 532 nm with an incident power of 10–30 mW, and a 50× objective with a numerical aperture of 0.5. For Raman measurements (see Section S12), we use an integration time of 600 s with a standard 2400 lines/mm grating.

### Estimating laser spot size

To determine the laser spot diameter, we employ a calibrated charge-coupled device (CCD) camera positioned in front of the objective lens to capture the original laser spot, yielding a 1/e^2^ diameter of *D*_original_ = 2124 μm. Considering a wavelength (*λ*) of 532 nm, a 50× objective lens focal length (*f*) of 4 mm, and a propagation *M*^2^ value of 1.1, we further calculate the 1/e^2^ diameter of the projected laser spot as $d = 4{{M}^2}\lambda f/\pi {{D}_{\rm {original}}} = 1.4$  ${\mathrm{\mu m}}$.

## Supplementary Material

nwae248_Supplemental_File
