# Supplementary material for: Investigating thermal properties of 2D non-layered material using a NEMS-based 2-DOF approach towards ultrahigh-performance bolometer
Source: Natl Sci Rev. 2024 Jul 17;11(10):nwae248. doi: 10.1093/nsr/nwae248 (PMC11409892; doi:10.1093/nsr/nwae248)
Supplement: nwae248_Supplemental_File [file nwae248_supplemental_file.pdf]

Supplementary Information

Investigating thermal properties of 2D non-layered material using a NEMS-based 2-DOF approach towards ultrahigh-performance bolometer

Luming Wang[1,#], Song Wu[1,#], Zejuan Zhang[1,#], Jiankai Zhu[1,#,*], Luwei Zou[2], Bo Xu[1], Jiaqi Wu[1], Junzhi Zhu[1], Fei Xiao[1], Chenyin Jiao[1], Shenghai Pei[1], Jiaze Qin[1], Yu Zhou[2*], Juan Xia[1*], Zenghui Wang[1,3*]

[1]*Institute of Fundamental and Frontier Sciences, University of Electronic Science and Technology of China, Chengdu 610054, China*
[2]*School of Physics, Hunan Key Laboratory of Nanophotonics and Devices, Central South University, Changsha 410083, China.*
[3]*State Key Laboratory of Electronic Thin Films and Integrated Devices, University of Electronic Science and Technology of China, Chengdu 611731, China*

[#]These authors contributed equally to this work [*]Corresponding authors

Section S1. Analysis of Optical Reflectance and Responsivity in Interferometric Motion Detection

Section S2. Analysis of Optical Absorbance in the Drumhead of NEMS Resonator

Section S3. Thermal Flux from Laser Heating

Section S4. Quantitative Analysis of Resonance Frequency in Circular Drumhead Resonators

Section S5. Extracting Young's Modulus and Thermal Expansion Coefficient *via* Analyzing Resonance Frequency

Section S6. Analysis of Laser Heating-Induced Frequency Tuning

Section S7. Discussion on Resolving Capability of the 2-DOF Approach

Section S8. Estimation of Uncertainty in Thermal Property Extraction

Section S9. Mapping of Resonance Response

Section S10. Benchmarking the Responsivity of 2D Resonant NEMS Bolometers

Section S11. Design for Resonant NEMS Bolometers

Section S12. Raman Shift Response to Laser Power and Laser Position

Section S13. Frequency Stability of the 2D β-In_2S_3 Resonator

Section S14. Reproducibility of the Resonance Measurements

Section S1. Analysis of Optical Reflectance and Responsivity in Interferometric Motion Detection

By employing ultrasensitive optical interferometry, we are able to detect the infinitesimal motion in the NEMS resonators, and the displacement sensitivity can reach fm/Hz$^{1/2}$ level[1]. For a typical 2D drumhead resonator (the inset in Figure S1(a)), a laser beam is focused on the device, with the reflected laser from the different interfaces interfering with each other. When the resonator is driven into motion, the varying vacuum gap modulates the reflected laser intensity. Thus, by transducing and analyzing the interferometric signal, the device's mechanical motion can be measured.

Analysis of the interferometry process gives the reflectance *R*, *i.e.*, the ratio of total reflected light intensity to incident light intensity, as[2]:

$$R = \frac{I_{\text{interferometry}}}{I_{\text{incident}}} = \left| \frac{\boldsymbol{r}_1 + \boldsymbol{r}_2 e^{-2i\boldsymbol{\varphi}_1} + \boldsymbol{r}_3 e^{-2i(\boldsymbol{\varphi}_1+\varphi_2)} + \boldsymbol{r}_1\boldsymbol{r}_2\boldsymbol{r}_3 e^{-2i\varphi_2}}{1 + \boldsymbol{r}_1\boldsymbol{r}_2 e^{-2i\boldsymbol{\varphi}_1} + \boldsymbol{r}_1\boldsymbol{r}_3 e^{-2i(\boldsymbol{\varphi}_1+\varphi_2)} + \boldsymbol{r}_2\boldsymbol{r}_3 e^{-2i\varphi_2}} \right|^2, \qquad \text{(Eq. S1)}$$

where $\boldsymbol{r}_1$, $\boldsymbol{r}_2$ and $\boldsymbol{r}_3$ represent reflection coefficients for the vacuum-In_2S_3, In_2S_3-vacuum, and vacuum-silicon interfaces, respectively:

$$\boldsymbol{r}_1 = \frac{n_{\text{vacuum}} - \boldsymbol{n}_{\text{In}_2\text{S}_3}}{n_{\text{vacuum}} + \boldsymbol{n}_{\text{In}_2\text{S}_3}}, \quad \boldsymbol{r}_2 = \frac{\boldsymbol{n}_{\text{In}_2\text{S}_3} - n_{\text{vacuum}}}{\boldsymbol{n}_{\text{In}_2\text{S}_3} + n_{\text{vacuum}}}, \quad \boldsymbol{r}_3 = \frac{n_{\text{vacuum}} - \boldsymbol{n}_{\text{silicon}}}{n_{\text{vacuum}} + \boldsymbol{n}_{\text{silicon}}}, \qquad \text{(Eq. S2)}$$

while $\boldsymbol{\varphi}_1$ and φ_2 are the associated phase shifts, originating from the optical path difference:

$$\boldsymbol{\varphi}_1 = \frac{2\pi}{\lambda_0} \boldsymbol{n}_{\text{In}_2\text{S}_3} t, \quad \varphi_2 = \frac{2\pi}{\lambda_0} n_{\text{vacuum}} d_{\text{vacuum}}. \qquad \text{(Eq. S3)}$$

Here, $n_{\text{vacuum}}=1$, and $\boldsymbol{n}_{\text{In2S3}}$ and $\boldsymbol{n}_{\text{silicon}}$ stand for the complex refractive indexes of In_2S_3 and silicon, respectively. λ_0 is the laser wavelength, t is the In_2S_3 membrane thickness, and d_{vacuum} represents the vacuum gap depth.

Using Device #1 as an example, we plot its reflectance *R* as a function of the vacuum gap in Figure S1(a). The refractive indexes used are given in Table S1.

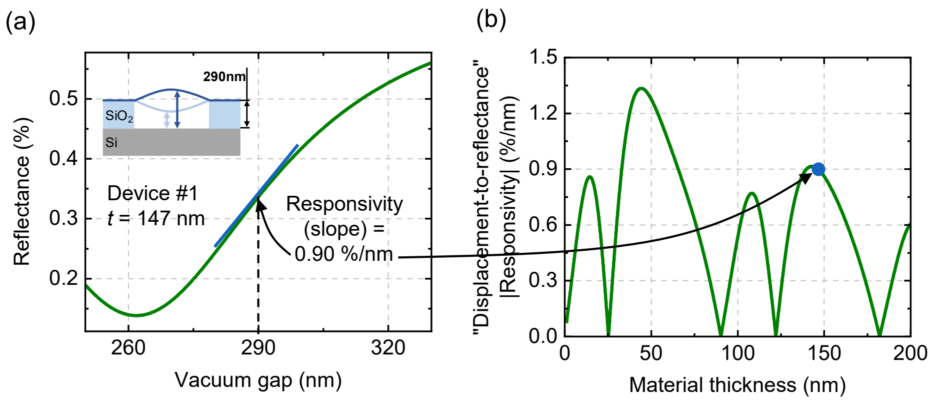

Figure S1. Analysis of optical reflectance and responsivity. (a) Calculation of the optical reflectance as a function of vacuum gap for Device #1 using a 532 nm laser. The inset illustrates a schematic of the device's cross section. **(b)** Calculation of the "displacement-to-reflectance" responsivity as a function of material thickness. Calculated values for Device #1 is indicated in both panels.

The "displacement-to-reflectance" responsivity $\Re$ is defined as $\Re = \partial R/\partial d_{\mathrm{vacuum}}$, and we extract the responsivity of Device #1 to be 0.90 %/nm. A higher responsivity allows more sensitive detection of device motion. We also plot the responsivity as a function of material thickness in Figure S1(b).

Furthermore, we investigate the influence of laser wavelength on responsivity, comparing the result obtained with two commonly used wavelengths, 532 nm and 633 nm, as shown in Figures S2(a)−(b). Both lasers offer decent responsivity for the devices of interest. Given that the 532 nm laser provides higher heating efficiency, it is used for experiments in this study (with more discussion in Section S2).

We also consider another type of substrate structure, in which the oxide is not entirely etched and a thickness of 40 nm of SiO_2 remains (the inset in Figure S2(b)), it can also be analyzed using a similar method. The interference process for this structure involves an additional layer, and its reflectance is given by[3]:

$$R = \left| \frac{\begin{array}{l} \boldsymbol{r}_1 + \boldsymbol{r}_2 e^{-2i\varphi_1} + \boldsymbol{r}_3' e^{-2i(\varphi_1+\varphi_2)} + \boldsymbol{r}_4' e^{-2i(\varphi_1+\varphi_2+\varphi_3)} + \boldsymbol{r}_1\boldsymbol{r}_2\boldsymbol{r}_3' e^{-2i\varphi_2} \\ + \boldsymbol{r}_1\boldsymbol{r}_2\boldsymbol{r}_4' e^{-2i(\varphi_2+\varphi_3)} + \boldsymbol{r}_1\boldsymbol{r}_3\boldsymbol{r}_4' e^{-2i\varphi_3} + \boldsymbol{r}_2\boldsymbol{r}_3'\boldsymbol{r}_4' e^{-2i(\varphi_1+\varphi_3)} \end{array}}{\begin{array}{l} 1 + \boldsymbol{r}_1\boldsymbol{r}_2 e^{-2i\varphi_1} + \boldsymbol{r}_1\boldsymbol{r}_3' e^{-2i(\varphi_1+\varphi_2)} + \boldsymbol{r}_1\boldsymbol{r}_4' e^{-2i(\varphi_1+\varphi_2+\varphi_3)} + \boldsymbol{r}_2\boldsymbol{r}_3' e^{-2i\varphi_2} + \\ \boldsymbol{r}_2\boldsymbol{r}_4' e^{-2i(\varphi_2+\varphi_3)} + \boldsymbol{r}_3\boldsymbol{r}_4' e^{-2i\varphi_3} + \boldsymbol{r}_1\boldsymbol{r}_2\boldsymbol{r}_3'\boldsymbol{r}_4' e^{-2i(\varphi_1+\varphi_3)} \end{array}} \right|^2, \quad \text{(Eq. S4)}$$

where $\boldsymbol{r}_3'$ and $\boldsymbol{r}_4'$ represent reflection coefficients for the vacuum-SiO_2 and SiO_2-silicon

interfaces respectively, and φ_3 denotes phase shift for the residual oxide domain:

$$r_3' = \frac{n_{\text{vacuum}} - n_{\text{SiO}_2}}{n_{\text{vacuum}} + n_{\text{SiO}_2}}, \quad r_4' = \frac{n_{\text{SiO}_2} - n_{\text{silicon}}}{n_{\text{SiO}_2} + n_{\text{silicon}}}, \quad \varphi_3 = \frac{2\pi}{\lambda_0} n_{\text{SiO}_2} d_{\text{SiO}_2}. \qquad \text{(Eq. S5)}$$

Figures S2(c)−(d) compare the responsivity of the two substrate structures, and we observe that the substrate structure with oxide fully etched exhibits a higher peak responsivity.

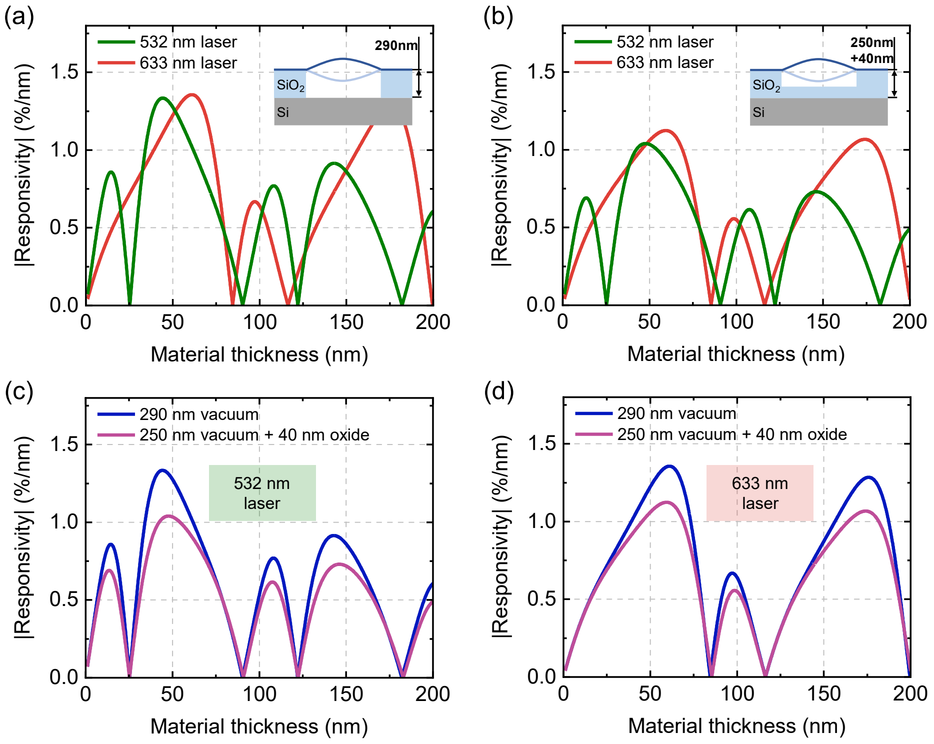

Figure S2. Comparison of optical interferometric responsivity across various laser wavelengths and device structures. (a-b) Responsivity of different laser wavelengths for microcavities on substrates **(a)** without and **(b)** with residual silicon oxide. Insets provide schematics of the substrate cross-sections. **(c-d)** Responsivity of different device structures, *i.e.*, different interferometry conditions, using **(c)** 532 nm and **(d)** 633 nm laser.

Table S1. Refractive indexes used in our interferometry calculations

Material	532 nm laser	633 nm laser
***β*-In$_2$S$_3$** [4]	2.81 – 0.14i	2.74 – 0.018i
**SiO$_2$** [5]	1.46 – 0.0019i	1.46 – 0.0016i
**Si** [6]	4.15 – 0.044i	3.88 – 0.019i

Section S2. Analysis of Optical Absorbance in the Drumhead of NEMS Resonator

The absorbance A of the drumhead in the NEMS resonator can be calculated by:

$$A = 1 - R - T. \quad \text{(Eq. S6)}$$

Here, R and T stand for the reflectance and transmittance of the device, defined as the ratio of total reflected light intensity to incident light intensity and the ratio of total transmitted light intensity to incident light intensity, respectively. Note that the term "transmittance" here refers to the part of the light passing through the device structure (less the substrate) rather than the 2D layer alone, and therefore numerically it equals the absorbance of the substrate.

The reflectance of the device structure featuring a fully etched micro-cavity is presented in Equation S1. Its transmittance can be expressed by[3]:

$$T = \left|\frac{\boldsymbol{n}_{\text{silicon}}}{\boldsymbol{n}_{\text{vacuum}}}\right| \left|\frac{\boldsymbol{t}_1\boldsymbol{t}_2\boldsymbol{t}_3 e^{-i(\varphi_1+\varphi_2)}}{1+\boldsymbol{r}_1\boldsymbol{r}_2 e^{-2i\varphi_1} + \boldsymbol{r}_1\boldsymbol{r}_3 e^{-2i(\varphi_1+\varphi_2)} + \boldsymbol{r}_2\boldsymbol{r}_3 e^{-2i\varphi_2}}\right|^2, \quad \text{(Eq. S7)}$$

where $\boldsymbol{t}_1$, $\boldsymbol{t}_2$ and $\boldsymbol{t}_3$ represent transmission coefficients for the vacuum-In_2S_3, In_2S_3-vacuum and vacuum-silicon interfaces respectively:

$$\boldsymbol{t}_1 = \frac{2n_{\text{vacuum}}}{n_{\text{vacuum}} + \boldsymbol{n}_{\text{In}_2\text{S}_3}}, \quad \boldsymbol{t}_2 = \frac{2\boldsymbol{n}_{\text{In}_2\text{S}_3}}{\boldsymbol{n}_{\text{In}_2\text{S}_3} + n_{\text{vacuum}}}, \quad \boldsymbol{t}_3 = \frac{2n_{\text{vacuum}}}{n_{\text{vacuum}} + \boldsymbol{n}_{\text{silicon}}}. \quad \text{(Eq. S8)}$$

We plot the optical reflectance, transmittance, and consequent absorbance as functions of material thickness in Figure S3(a).

Considering a device structure containing a residual layer of silicon oxide, its reflectance R is given by Equation S4, and its transmittance can be expressed as[7]:

$$T = \left|\frac{\boldsymbol{n}_{\text{silicon}}}{\boldsymbol{n}_{\text{vacuum}}}\right| \left|\frac{\boldsymbol{t}_1\boldsymbol{t}_2\boldsymbol{t}_3'\boldsymbol{t}_4' e^{-i(\varphi_1+\varphi_2+\varphi_3)}}{\begin{array}{c}1+\boldsymbol{r}_1\boldsymbol{r}_2 e^{-2i\varphi_1} + \boldsymbol{r}_1\boldsymbol{r}_3' e^{-2i(\varphi_1+\varphi_2)} + \boldsymbol{r}_1\boldsymbol{r}_4' e^{-2i(\varphi_1+\varphi_2+\varphi_3)} + \boldsymbol{r}_2\boldsymbol{r}_3' e^{-2i\varphi_2} + \\ \boldsymbol{r}_2\boldsymbol{r}_4' e^{-2i(\varphi_2+\varphi_3)} + \boldsymbol{r}_3\boldsymbol{r}_4' e^{-2i\varphi_3} + \boldsymbol{r}_1\boldsymbol{r}_2\boldsymbol{r}_3'\boldsymbol{r}_4' e^{-2i(\varphi_1+\varphi_3)}\end{array}}\right|^2, \quad \text{(Eq. S9)}$$

where the transmission coefficients $\boldsymbol{t}_3'$ and $\boldsymbol{t}_4'$ are given by:

$$t_3' = \frac{2n_{\text{vacuum}}}{n_{\text{vacuum}} + n_{\text{SiO}_2}}, \quad t_4' = \frac{2n_{\text{SiO}_2}}{n_{\text{SiO}_2} + n_{\text{silicon}}}. \qquad \text{(Eq. S10)}$$

We plot the optical coefficients for this structure in Figure S3(b). The overall absorbance curve closely aligns with that in Figure S3(a), albeit exhibiting a small decrease. In addition to interferometric analysis, we utilize FEM to undertake numerical simulations for validation. The FEM simulation results, plotted as solid lines in Figure S3, show good agreement with the theoretical results discussed above.

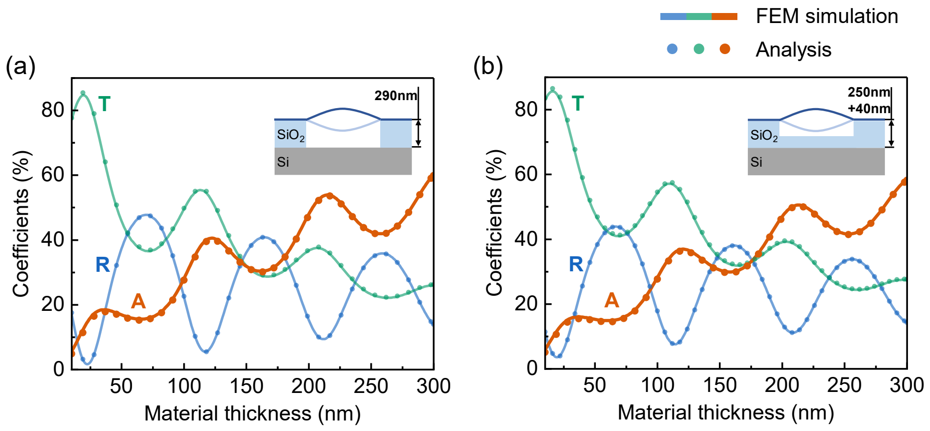

Figure S3. Comparison of optical transmittance, reflectance, and absorbance as a function of drumhead thickness for the two device structures. 532 nm wavelength is used. Solid lines illustrate wave-optics FEM simulation results, while solid circles indicate data derived from the equations. Green, blue, and orange curves represent transmittance, reflectance, and absorbance, respectively.

The choice of laser wavelength affects the absorbance and, consequently, the overall performance of this approach. According to the calculations, the complex refractive index is a key factor in determining the optical absorption. For β-In_2S_3, its refractive index can vary substantially in the visible optical band. We evaluate the performance of two commonly used laser wavelengths, 532 nm and 633 nm, and plot their optical absorbance and simulated frequency response (see more discussion in the following sections) in Figure S4.

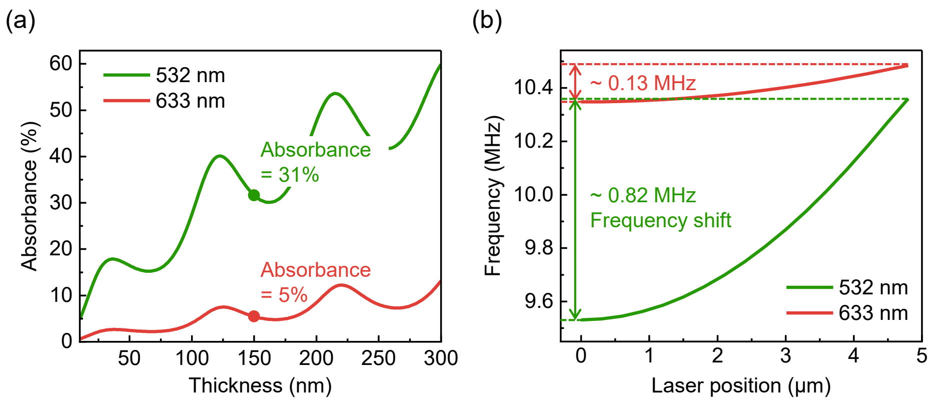

Figure S4. Numerical analysis of the influence of laser wavelength selection. (a) Calculated optical absorbance *vs*. β-In_2S_3 thickness, assuming a fully-etched substrate is used. **(b)** Calculated frequency response *vs*. laser position (the "position" curve) by using specific absorbance values denoted by solid dots in (a), calculated using a 0.5 mW laser power, for a 150 nm thick and 10 μm diameter drumhead device.

Section S3. Thermal Flux from Laser Heating

We assume that the laser spot follows a Gaussian distribution radially in terms of light intensity, with a device radius w = 0.7 μm, defined as the radius at which the intensity falls to $1/e^2$ of its maximum value. The peak intensity I_0 can be calculated by limiting the total power within a circle of radius r:

$$I_0 = \lim_{r\to 0} \frac{P_0[1-e^{\frac{-2r^2}{w^2}}]}{\pi r^2} = \frac{2P_0}{\pi w^2}, \qquad \text{(Eq. S11)}$$

where P_0 represents the total power of the laser beam. Consequently, for a Gaussian distributed laser spot centered at position (x_0, y_0), the thermal source flux (induced by the laser) at any point (x, y) in the plane can be expressed as:

$$I(x,y) = I_0 \exp(\frac{-2((x-x_0)^2+(y-y_0)^2)}{w^2}). \qquad \text{(Eq. S12)}$$

As an example, we consider a laser beam positioned at the center of the drumhead with a laser power of 1 mW, from the above equations we can determine the thermal source flux induced by the laser beam, with results shown in Figure S5.

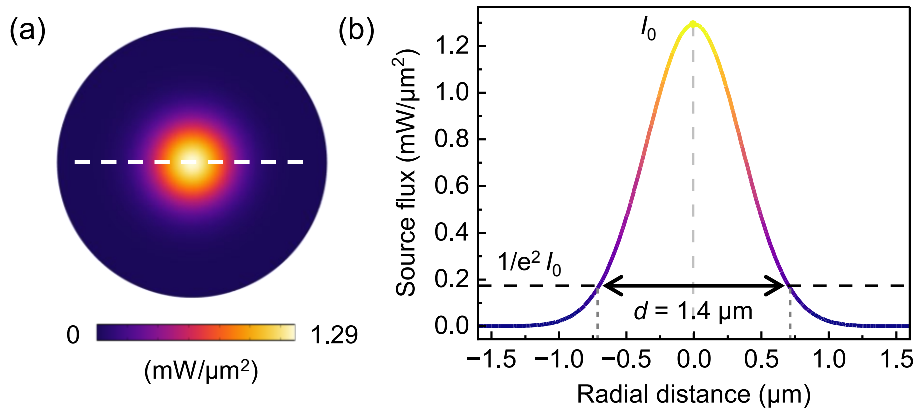

Figure S5. Illustration on laser-induced thermal source flux. (a) 2D distribution of thermal source flux for a Gaussian laser beam with a spot size (diameter) of 1.4 μm. **(b)** Profile along the white dashed line in (a).

Section S4. Quantitative Analysis of Resonance Frequency in Circular Drumhead Resonators

In this section, we provide details for theoretical analysis for determining the resonance frequency of circular drumhead resonators. For circular drumhead resonators with both bending rigidity and non-zero tension, the resonance frequency of the *mn*-th mode (*m* and *n* stand for the number of interior nodal circles and nodal diameters, respectively) can be calculated as[8,9]:

$$f_{mn} = \frac{k_2^{mn} a}{2\pi} \sqrt{\frac{D}{\rho a^4} \left[\frac{\gamma a^2}{D} + (k_2^{mn} a)^2 \right]}. \qquad \text{(Eq. S13)}$$

In this expression, k_2^{mn} denotes a mode-dependent parameter which can be determined numerically, $a = d/2$ is the radius, ρ is the areal mass density (kg/m^2), γ is the in-plane tension (N/m), and D is the bending rigidity:

$$D = \frac{E_Y t^3}{12(1-v^2)}. \qquad \text{(Eq. S14)}$$

Here, E_Y is Young's modulus, v is Poisson's ratio and t denotes the material thickness. In all the analysis for β-In_2S_3, we employ a mass density of $\rho_{3D} = \rho/t = 4613$ kg/m^3 [10], and a Poisson's ratio of 0.257 [11]. Note that the Poisson's ratio for β-In_2S_3 is not available in the literature. Therefore, the value from Si_2N_2O, a material which has a similar crystalline structure to β-In_2S_3, is used for the calculation. It is known that the value of Poisson's ratio (within a reasonable range) has very little influence on the calculated frequency[9].

For the parameter k_2^{mn}, often the following double-exponential asymptotic function[12] is used to approximate the dependency of k_2^{mn} on device parameters:

$$(k_2^{mn} a)^2 = \alpha_{mn} + (\beta_{mn} - \alpha_{mn}) \exp\left\{ -\eta_{mn} \exp\left[\delta_{mn} \ln\left(\frac{\gamma a^2}{D}\right) \right] \right\}. \qquad \text{(Eq. S15)}$$

It is noteworthy to discuss the numerical error of this approximation. As reported previously[12], this approximation introduces a maximum error of about 3.8%. To examine the influence of this error on our frequency analysis, we take a closer look at

the exact solution[8] for k_2^{mn}:

$$\begin{cases} \begin{vmatrix} J_m(k_2a) & I_m(k_1a) \\ (k_2a)J_m^{'}(k_2a) & (k_1a)I_m^{'}(k_1a) \end{vmatrix} = 0 \\ (k_1a)^2 - (k_2a)^2 = \dfrac{\gamma a^2}{D} \end{cases} . \qquad \text{(Eq. S16)}$$

Here, J_m and I_m denote the m-th order cylindrical and modified cylindrical Bessel functions, respectively, while J'_m and I'_m are their respective derivatives.

For the determinant in Equation S16, the slope becomes increasingly steep near its root, which is attributed to the multiplication of the Bessel functions. This causes the approximation to possess numerical error. Here we use the exact method to solve k_2^{mn}, then compare them to evaluate whether it is still viable to use the approximation method in this work.

In Figure S6(a), we present the calculated device frequency using both the approximation in Eq. S15 (dashed lines) and the numerical method in solving Eq. S16 (solid lines), as functions of device thickness. Figure S6(b) compares the two calculated frequencies when varying device tension for a device with the dimension of Device #1 in the Main Text.

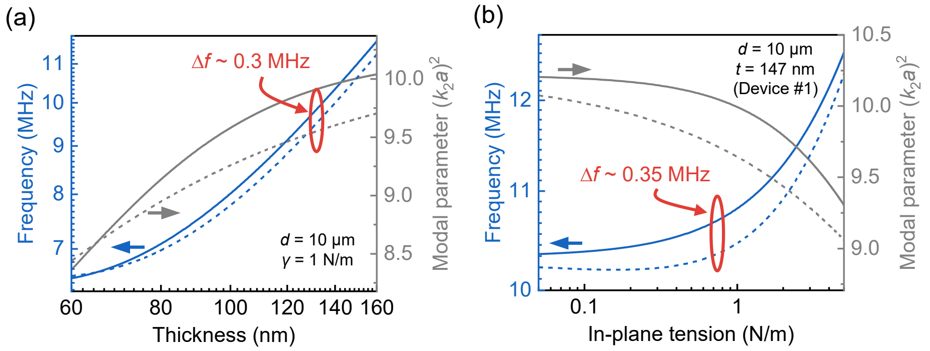

Figure S6. Illustration of discrepancies between the numerical and the approximation methods in calculating k_2^{mn} and device frequency. Solid lines denote results calculated using the exactly numerical solutions to Eq. S16, while dashed lines denote results from the approximation expression in Eq. S15. Red ovals indicate the maximum discrepancies.

We observe that the maximum discrepancy between the approximation method and the numerical method is about 0.35 MHz, as indicated by the red ovals in the

plots. As a comparison, during the experiments of sweeping the laser position, the overall frequency shift observed (the difference between frequencies when the laser is centrally positioned compared to when it is at the edge) amounts to merely ~0.5 MHz (Main Text Figure 1). This suggests that the error induced by using the approximation method could be comparable to the observed thermal frequency shift in our experiments. Therefore, it is necessary to use the exact method (numerically solving Equation S16) to calculate the theoretical frequency, which is what we used in this study.

Section S5. Extracting Young's Modulus and Thermal Expansion Coefficient *via* Analyzing Resonance Frequency

To extract the Young's modulus, we measured multiple devices to determine the frequency scaling law. Figure S7 shows the measured fundamental mode resonance frequency f_0 from multiple devices (device details in Table S2), plotted as a function of device thickness, from which we extract the Young's modulus of β-In_2S_3 crystal as E_Y = 60 GPa by optimizing fitting between the theoretical model (Equation S13) and the experimental results. This analysis also suggests a built-in tension on the order of 1 N/m (the shaded area in Figure S7), consistent with values commonly found in devices fabricated using similar techniques[9,13].

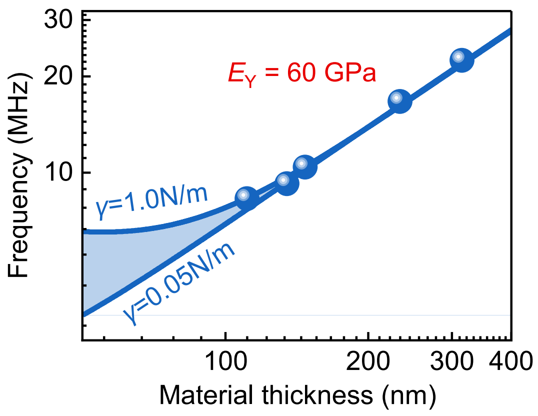

Figure S7. Extraction of Young's modulus. Spheres denote the measured resonance frequency of devices, and lines and areas denote the calculated frequency range with different initial surface tension γ_0.

To measure the thermal expansion coefficient, the device is mounted in a cryostat, which can be cooled to 77 K using liquid nitrogen. We measure the resonance frequency of the device as a function of temperature (data taken every 5 K), with results shown in Figure S8(a). A PID-regulated temperature controller is employed to stabilize the temperature.

To analyze the thermal expansion from the temperature-dependent device frequency, we first convert the thermal-induced tension into tensile strain[14]:

$$\gamma_{\text{total}} = \frac{2E_{\text{Y}}t\varepsilon_{\text{total}}}{3(1-\nu)}, \qquad \text{(Eq. S17)}$$

where γ_{total} denotes the total in-plane tension, $\varepsilon_{\text{total}}$ denotes the total tensile strain, and E_{Y}, v, t stand for Young's modulus, Poisson's ratio, and material thickness, respectively. From the device frequency we then calculate the device tension and strain.

Figure S8(b) (left axis) shows a good linear relationship between tensile strain and temperature. This linearity indicates that the thermal expansion coefficient of β-In_2S_3 remains mostly constant in this temperature range. By fitting the strain response using the equation $\varepsilon_{total} = \varepsilon_0 - \alpha(T - T_0)$, where α stands for the thermal expansion coefficient and T_0 denotes the room temperature, we extract an α value of 10 ppm for 2D β-In_2S_3 crystal within this temperature range.

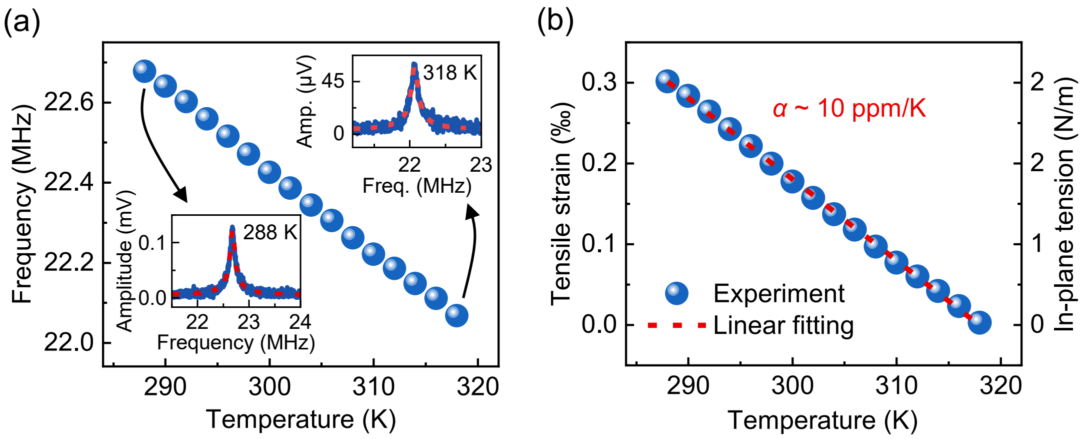

Figure S8. Determination of thermal expansion coefficient. (a) Temperature-dependent resonance frequency of an In_2S_3 resonator. Insets show the resonance spectra of the device under two temperature extremes. **(b)** The tensile strain and in-plane tension values *vs.* temperature, derived from the frequency response shown in **(a)**. The red dashed line represents a linear fit, yielding a thermal expansion coefficient α ~10 ppm/K.

Section S6. Analysis of Laser Heating-Induced Frequency Tuning

For a circular drumhead resonator with a material thickness of t and a suspended area of A, its thermally induced in-plane stress γ_{th} can be determined through an average surface temperature difference ΔT_{avg} as follows[14,15]:

$$\Delta T_{\mathrm{avg}} = \frac{1}{A}\iint_A \Delta T(x,y)\,\mathrm{d}x\mathrm{d}y, \qquad \text{(Eq. S18)}$$

$$\gamma_{\mathrm{th}} = \frac{t}{A}\iint_A \frac{2}{3}\sigma\,\mathrm{d}x\mathrm{d}y = -\frac{2tE_{\mathrm{Y}}\alpha\Delta T_{\mathrm{avg}}}{3(1-v)}, \qquad \text{(Eq. S19)}$$

where σ represents the thermally induced stress per unit length, α represents the thermal expansion coefficient of the 2D β-In_2S_3, and E_{Y} and v are the Young's modulus and Poisson's ratio of the material. To simplify the analysis, we make an assumption that the Young's modulus E_{Y} remains relatively constant within the temperature range of interest, which is commonly assumed in such studies[16]. Taking into account the influence of thermal stress γ_{th}, the resonance frequency can be expressed as[9,12]:

$$f_0=\left(\frac{kd}{4\pi}\right)\sqrt{\frac{16D}{\rho_{2D}d^4}\left[\left(\frac{kd}{2}\right)^2+\frac{(\gamma_0+\gamma_{\mathrm{th}})d^2}{4D}\right]} \qquad \text{(Eq. S20)}$$

where k represents a modal parameter that is determined numerically, γ_0 is the initial surface tension without thermal stress (in N/m). The flexural rigidity D is given by $D = E_{\mathrm{Y}}t^3/\,[12(1-v^2)]$.

The above equations relate ΔT_{avg} to device frequency. Therefore, by experimentally measuring frequency, one can derive ΔT_{avg}. In order to relate ΔT_{avg} to the thermal properties, we numerically estimate ΔT_{avg} for a given laser illumination with a power of P_0 focused at position (x, y) by solving the following equations, in which the thermal properties are important parameters. This allows us to compare the calculated results with experiments and then extract the thermal parameters.

The surface temperature distribution of the 2D β-In_2S_3 resonator can be described by the following set of equations:

$$r^2 = (x - x_0)^2 + (y - y_0)^2$$

$$\kappa\left(\frac{\partial^2 T}{\partial x^2} + \frac{\partial^2 T}{\partial y^2} + \frac{\partial^2 T}{\partial z^2}\right) + \frac{2P_0 A_0}{\pi t w^2}\exp\left(\frac{-2r^2}{w^2}\right) = 0, \quad (0 \le r \le r_{\text{material}}) \quad \text{(Eq. S21)}$$

$$\boldsymbol{n}\left(\kappa\left(\frac{\partial^2 T}{\partial x^2} + \frac{\partial^2 T}{\partial y^2}\right)\right) - G_{\text{B}}\Delta T(x, y) = 0, \quad (r_{\text{suspended}} \le r \le r_{\text{material}})$$

In these equations, r is the distance from any point (x, y) on the material to the center of the laser spot at (x_0, y_0), P_0 is the total power of the laser, t is the material thickness, and A_0 is the absorbance of the drumhead in the NEMS resonator. The parameters κ is thermal conductivity of β-In_2S_3, G_B is interfacial thermal conductance between β-In_2S_3 and SiO_2, $\Delta T(x, y)$ stands for the temperature difference at the supported area, and $\boldsymbol{n}$ denotes the normal vector of the supported material facing SiO_2. The detailed temperature distribution $T(x, y, z)$ is calculated numerically using finite element model, and the ΔT_{avg} is then calculated by averaging $T(x, y, z)$ over the entire device area.

In our analysis, ΔT_{avg} values determined from the measured resonance frequency response are then fitted to ΔT_{avg} curves calculated from the laser power and laser position through the above equations, with κ and G_B as fitting parameters. By optimizing the fitting, thermal properties can be extracted, as shown in Figures 3 & 4 of the Main Text.

Section S7. Discussion on Resolving Capability of the 2-DOF Approach

The two distinct DOFs utilized in our study, laser position and laser power, offer the capability to quantitatively extract values for κ and G_B. Figure 3 of the Main Text showcases several such examples. Here we take a closer look at the κ and G_B resolving capabilities of the "position" and "power" curves, *i.e.*, measurements leveraging the two DOFs.

We first examine the "position" curve. Here we define a "frequency shift" Δf_0 as the absolute frequency difference between when the laser is at the center and at the edge of the device, which is essentially the vertical "elevation" of the "position" curve. Then we plot this frequency shift Δf_0, calculated as a function of κ and G_B, as a curved surface in the 3D space, shown in Figure S9(a). The resulting surface clearly curves up along the κ axis, while much less so along the G_B axis.

Next, we quantify how sensitive this frequency shift Δf_0 is to changes in ΔG_B and $\Delta\kappa$. Shown in Figure S9(b), which is taken from a vertical slice in (a), a $\Delta G_B/G_B$ variation of 174% (G_B from 1.1 to 15.8) results in merely 4% variation (from 0.602 MHz to 0.626 MHz) in the frequency shift value. This indicates that variation in G_B minimally impacts the frequency shift. In contrast, a $\Delta\kappa/\kappa$ variation of only 4% (κ from 4.88 to 5.06) can also lead to a 4% variation (from 0.602 MHz to 0.626 MHz) in frequency shift (Figure S9(c), taken from another vertical slice in (a)). This suggests that the frequency shift Δf_0 in the "position" curve is much more sensitive to κ than to G_B, and thus examining the "position" curve can be very effective in singling out κ (and pinpointing its value) while being minimally affected by the value of G_B.

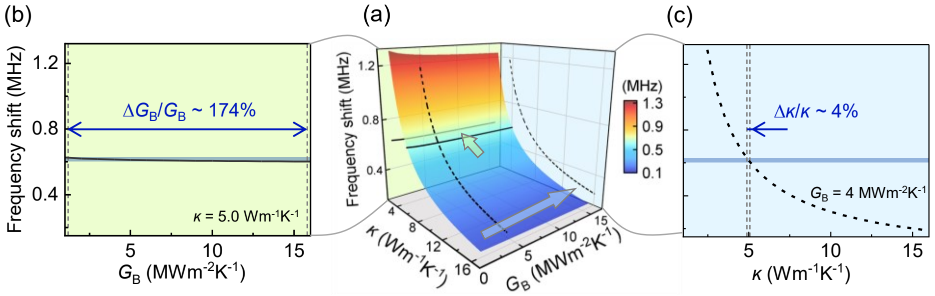

Figure S9. Examination of the frequency shift of the "position curve" under different

thermal property conditions. (a) 3D visualization of the frequency shift—defined as the absolute frequency difference while sweeping the laser position from center to edge—as a function of κ and G_B. Curves through a typical parameter pair (the black "cross" on the curved surface) are also projected onto the corresponding vertical planes, which are reproduced in **(b)** and **(c)** as 2D plots. Blue horizontal areas in (b) and (c) represent the measured frequency shift with 4% variation. Parameters from Device #1 are used in this calculation.

We further discuss the applicability of our approach through thermal resistance analysis. Thermal conduction inside the resonator can be modeled as heat traveling across two thermal resistances: the in-plane conduction resistance (R_{th1}), governed by κ, and the contact boundary resistance (R_{th2}), governed by G_B. We follow a simplified estimation method[15]:

$$\begin{cases} R_{th1} = \dfrac{\ln(d / d_{laser})}{2\pi\kappa t}, \\ R_{th2} = (\pi d G_B t)^{-1} \end{cases} \quad \text{(Eq. S22)}$$

where d is the device diameter, d_{laser} is the laser spot diameter, and t is the drumhead thickness. We categorize three distinct thermal resistance cases by selecting various pairs of thermal property parameters: κ-dominated, both-κ-and-G_B-dominated, and G_B-dominated. Calculated results are shown in Figure S10.

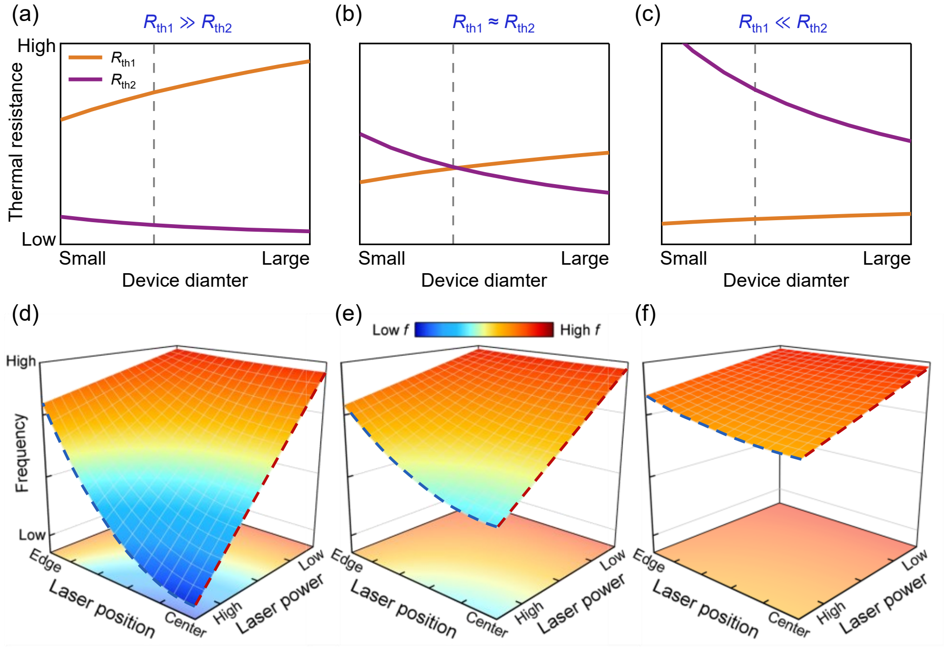

Figure S10. Laser heating-based frequency tuning across different thermal resistance cases. (a-c) Calculated thermal resistance values as functions of device diameter, when the system is **(a)** κ-dominated, **(b)** κ and G_B co-dominated, and **(c)** G_B-dominated. **(d-f)** Calculated device

frequency *vs.* laser position and laser power, corresponding to panels (a)-(c). The blue and red dashed curves at the edges depict the “position” and “power” curves, respectively, which are used to compare with the measurements. Different cases are illustrated: **(a & d)** $R_{th1} \gg R_{th2}$, **(b & e)** $R_{th1} \approx R_{th2}$, and **(c & f)** $R_{th1} \ll R_{th2}$.

This analysis reveals that regardless of the dominant factor, both frequency tuning methods—sweeping laser position and sweeping laser power—produce consistent response trends. Specifically, as shown by the red and blue dashed lines in Figure S10(d-f), the “position” curve consistently shows a curvilinear (with observable curvature) trend, whereas the “power” curve remains almost linear. With such differentiation, we expect that the 2-DOF approach can be applied to 2D materials with different thermal properties, regardless of whether the sample’s thermal transport is dominated by κ, G_B, or both.

Section S8. Estimation of Uncertainty in Thermal Property Extraction

To evaluate the quality of the fitting used for extracting the thermal parameters (κ, G_B), we examine the fitting parameter $R^2 = 1 - \frac{\text{residual sum of squares}}{\text{total sum of squares}}$ which is calculated by the fitting program. To estimate the error bars, we use values from fittings which result in R^2 values that are within 97% of the best achievable R^2 value, *i.e.*, $R^2 \geq 0.97 \times R^2_{\text{best-fit}}$, with one set of such results shown in Figure S11. The highest and lowest values that meet such criterion give the error bars in Figure 4 of the Main Text.

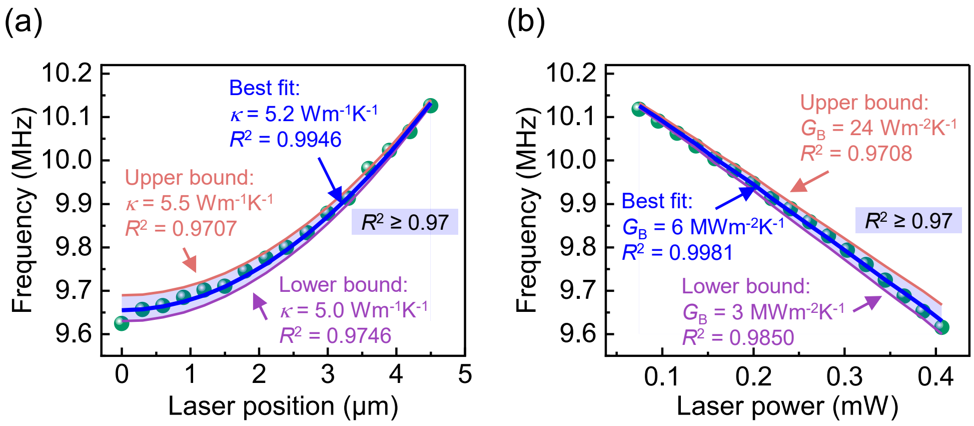

Figure S11. Illustration of uncertainty in extracting thermal properties. The measured and simulated frequency responses of **(a)** “position curve” and **(b)** “power curve” with the same data in Figures 4(a)-(b) of the Main Text. Green spheres are measurement data, blue lines are the best-fit curves, and the orange and purple lines denote the upper and lower bounds of the fitting, respectively.

Section S9. Mapping of Resonance Response

In this section, we present detailed spatial mapping (spectromicroscopy) results of our devices, including maps of resonance peak amplitude and maps of resonance frequency, as shown in Figures S12−S14, along with a summary of device performance in Table S2. Note that when analyzing position dependence of resonance frequency (the "position curve", such as Figure S12(e)), we average the responses from four orthogonal radial directions. This averaging process effectively reduces the effect of positioning errors in the measurements.

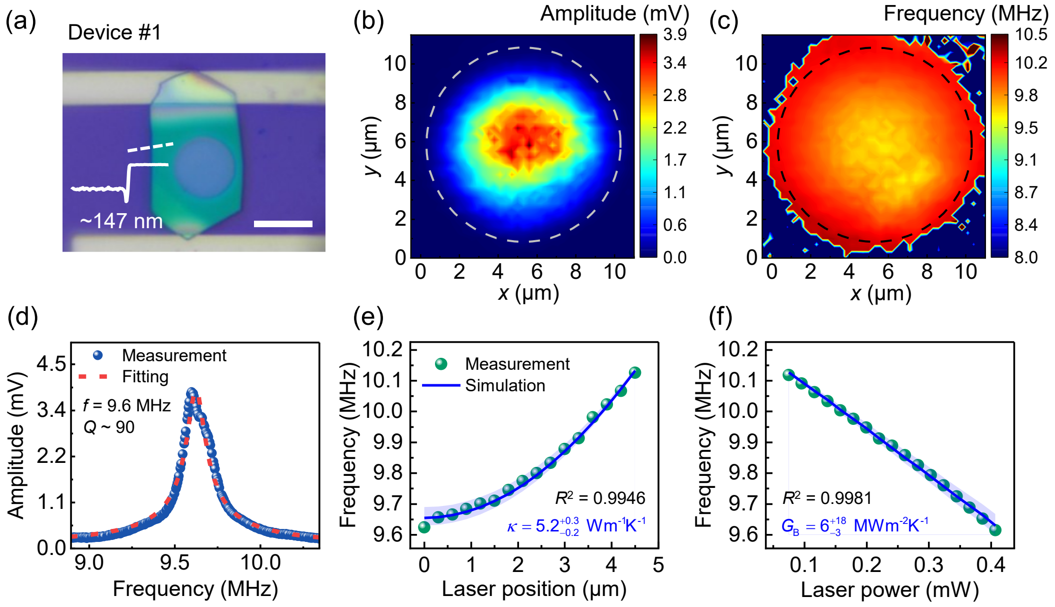

Figure S12 Experimental results for Device #1 with a thickness of ~147 nm. (a) Optical image of the device, with AFM height profile measured at the white dashed line. Scale bar: 10 μm. **(b-c)** Spatial mapping of **(b)** measured signal amplitude and **(c)** resonance frequency. Dashed circles indicate the boundaries of the suspended area. **(d)** Resonance spectrum measured at the center of the circular device. **(e-f)** Frequency responses as a function of **(e)** laser radial position and **(f)** laser power.

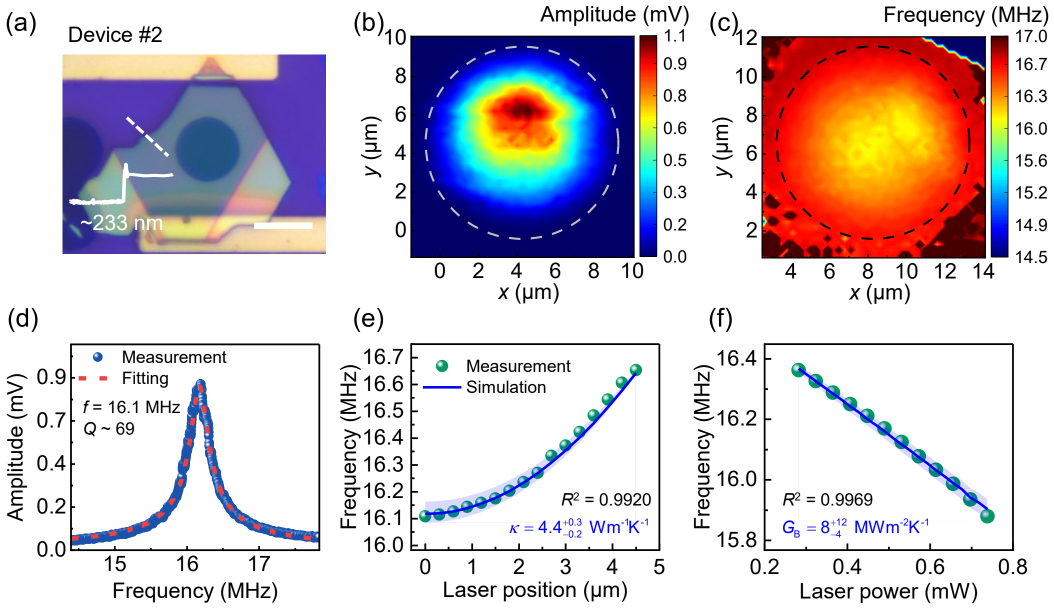

Figure S13. Experimental results for Device #2 with a thickness of ~233 nm. (a) Optical image of the device, with AFM height profile measured at the white dashed line. Scale bar: 10 μm. **(b-c)** Spatial mapping of **(b)** measured signal amplitude and **(c)** resonance frequency. Dashed circles indicate the boundaries of the suspended area. **(d)** Resonance spectrum measured at the center of the circular device. **(e-f)** Frequency responses as a function of **(e)** laser radial position and **(f)** laser power.

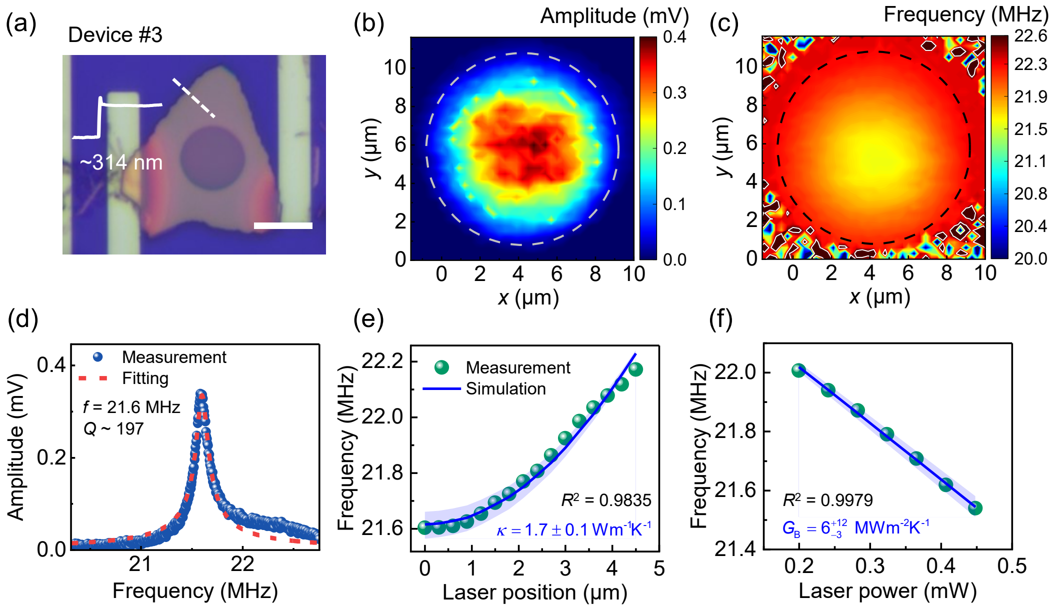

Figure S14. Experimental results for Device #3 with a thickness of ~314 nm. (a) Optical image of the device, with AFM height profile measured at the white dashed line. Scale bar: 10 μm. **(b-c)** Spatial mapping of **(b)** measured signal amplitude and **(c)** resonance frequency. Dashed circles indicate the boundaries of the suspended area. **(d)** Resonance spectrum measured at the center of the circular device. **(e-f)** Frequency responses as a function of **(e)** laser radial position and **(f)** laser power.

Table S2. Summary of Device Performance

Device #	Diameter	Thickness	Laser Power	*Laser spot at the center*		*Laser spot at the edge*	
				**Frequency**	***Q***	**Frequency**	***Q***
**1**	10 µm	~147 nm	448 µW	9.62 MHz	92	10.17 MHz	118
**2**	10 µm	~233 nm	741 µW	16.11 MHz	63	16.57 MHz	86
**3**	10 µm	~314 nm	448 µW	21.60 MHz	197	22.11 MHz	230
**4**	10 µm	~111 nm	240 µW	7.89 MHz	206	8.13 MHz	332
**5**	10 µm	~135 nm	240 µW	8.53 MHz	133	9.09 MHz	132

Section S10. Benchmarking the Responsivity of 2D Resonant NEMS Bolometers

We survey resonant NEMS bolometers with drumhead structure in the literature, with the results shown in Table S3 and Figure S15. The "laser-power-to-frequency-shift" ("power-to-frequency" in short) responsivity is the key performance metric for comparison.

Table S3. Summary of drumhead 2D resonant NEMS bolometers

Ref.	Year of Publication	Material	Lateral Dimension	Device Thickness	Initial Frequency	Wavelength	Responsivity (ppm/μW)
[17]	2012	Graphene	14 × 14 μm square drumhead	1 Layer	~4.02 MHz	568 nm	−47.17
					~5.15 MHz	633 nm	−20.80
[18]	2014	GaN-on-SOI	80 × 80 μm square plate	1.425 μm	~101 MHz	760~1000 nm	−142
[19]	2015	MoS_2	d = 5 μm circular drumhead	56 nm	21.8 MHz	633 nm	−41.16
					31.9 MHz		−15.29
[20]	2016	MoS_2	L = 4 μm beam	2 Layers	34.5 MHz	633 nm	−142
[15]	2018	Black P	d = 9 μm circular drumhead	85 nm	8.39 MHz	633 nm	~ −37.71
			d = 8 μm circular drumhead	30 nm	7.45 MHz		~ −285
[21]	2020	Black P	d = 4.6 μm circular drumhead	~30 nm	26.14 MHz	785 nm	−11.8
			d = 7 μm circular drumhead	~80 nm	14.3 MHz		−1.18
[1]	2023	$CaNb_2O_6$	d = 7 μm circular drumhead	69.1 nm	11.13 MHz	532 nm	~ −4.2
**This work**		**β-In_2S_3**	**d = 10 μm circular drumhead**	**~111 nm**	**7.19 MHz**	**532 nm**	**−447**

The responsivity values with "~" are estimated from the data.

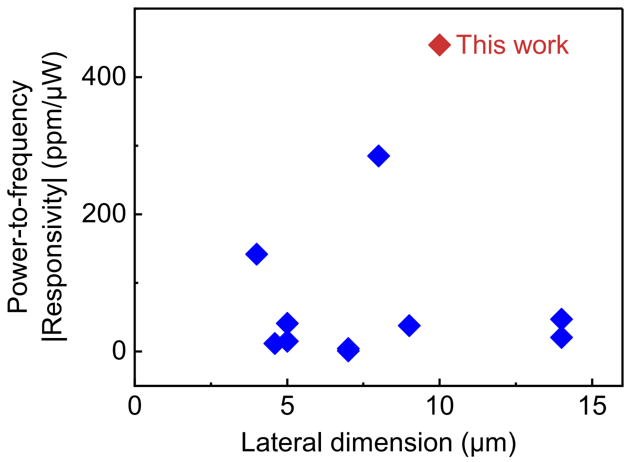

Figure S15. The power-to-frequency responsivity for 2D resonant NEMS bolometers reported in the literature. Data taken from Table S3.

Section S11. Design for Resonant NEMS Bolometers

We examine design guidelines for such resonant-NEMS-based bolometers, focusing on how device geometry affects the power-to-frequency responsivity.

Here we calculate the power-to-frequency responsivity $\Re_{\text{P-}f} = \frac{\partial f / f_0}{\partial P_{\text{laser}}}$, assuming a laser power of 200 μW. Considering the mostly linear response of the bolometer's frequency response to laser power in this range, the choice of laser power should not significantly affect the value of $\Re_{\text{P-}f}$. Results from numerical analyses are shown in Figure S16. The results suggest that in general, larger (laterally) and thinner devices exhibit higher responsivity, which is consistent with the expectation that larger heat resistance in such devices leads to slower heat dissipation and thus more pronounced heating effect for any given laser power.

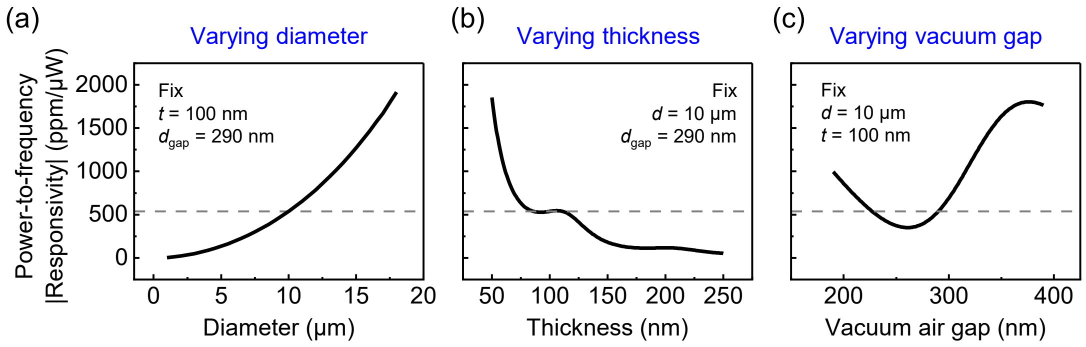

Figure S16. Estimating performance for resonant-NEMS-based bolometers of various device geometries. Theoretical power-to-frequency responsivity of In_2S_3 resonators are shown for **(a)** varying device diameter, **(b)** varying material thickness, and **(c)** varying vacuum air gap. The horizontal dashed lines intercept the three curves with the same device geometry: d = 10 μm, t = 100 nm, and d_{gap} = 290 nm. Calculations are based on an initial tension of 0.5 N/m. Other parameters remain consistent with those detailed in the Main Text.

Section S12. Raman Shift Response to Laser Power and Laser Position

Raman spectroscopy can also be used to extract thermal properties in 2D materials. In order to evaluate the capability of such Raman technique in extracting the thermal properties of the samples used in this study, we perform Raman measurements for the same β-In_2S_3 device while varying both laser position and laser power. The Raman spectra measured for Device #1 is shown in Figure S17.

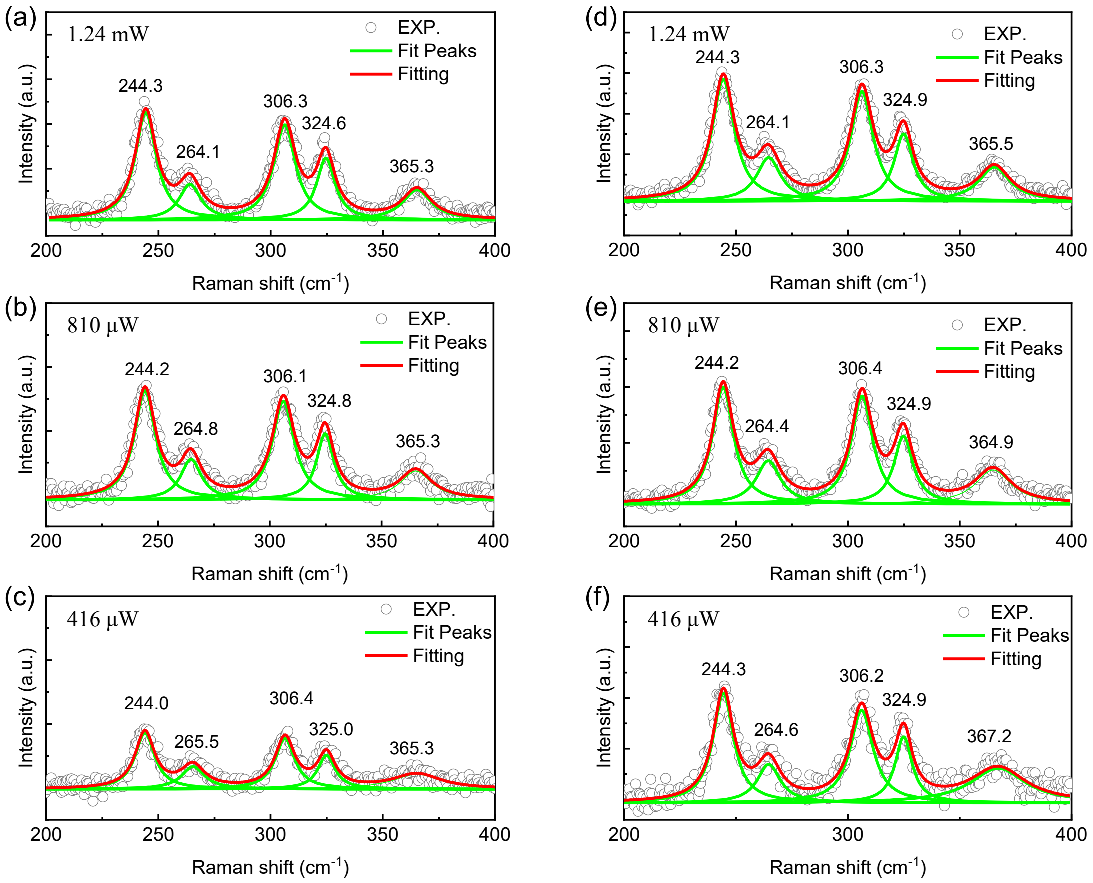

Figure S17. Raman spectra with various laser heating conditions. Measured (white spheres) Raman spectra with laser positioned **(a-c)** at the center and **(d-f)** at the edge of the suspended area of Device #1. The laser power used is labeled at the upper left corner of each panel. Red curves represent the accumulated fitting, and green curves show fittings for individual Raman peaks.

From these data we observe that varying laser power results in very limited shifts in Raman peak frequency, regardless of the laser position (at device center or edge), insufficient to extract thermal properties for this non-layered material. From Figure S18, we estimate that the maximum power-to-frequency responsivity of the Raman peak is 0.0047 ppm/μW. In comparison, the power-to-frequency responsivity of the

mechanical resonance response is −447 ppm/μW, ~1 million times more responsive.

In addition, the ability of a spectrometer to resolve Raman peak shift is typically worse than 0.5 cm^{-1}, which corresponds to a power sensitivity of ~242 μW. In comparison, the measured frequency stability (see Section S13 for more details) is ~25.23 ppm, which corresponds to a power sensitivity of ~0.11 μW, >2000 times more sensitive.

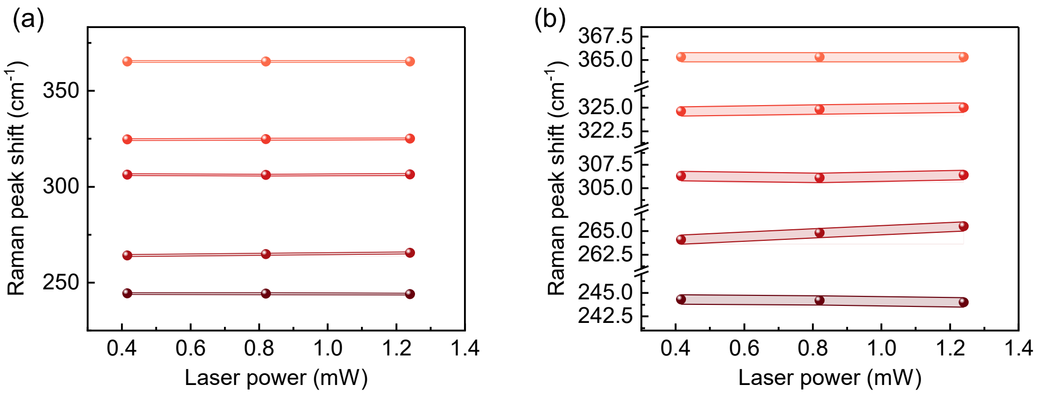

Figure S18. Raman shift in response to laser power. (a) Raman peak shift *vs.* laser power. Data taken from Figure S17 left panel. **(b)** Zoom-in (in vertical axis) of (a). The colored areas correspond to the resolution capability (±0.5 cm^{-1}) of our spectrometer. Measurement performed when the laser spot is positioned at the device center (which should generate the strongest laser heating effect).

Section S13. Frequency Stability of the 2D β-In_2S_3 Resonator

We characterize the frequency stability of the 2D β-In_2S_3 Resonator by monitoring its frequency over extended time. We calculate the root-mean-squared (RMS) fractional frequency shift (variation) from such measurement[22]:

$$\langle \delta f_0 / f_0 \rangle_\tau \cong \left[\frac{1}{N-1} \sum_{i=1}^{N} \left(\frac{\overline{f_i} - f_0}{f_0} \right)^2 \right]^{\frac{1}{2}}, \quad \text{(Eq. S23)}$$

where τ is the averaging time, f_0 is the average of all resonance frequency values, and $\overline{f_i}$ is the averaged frequency in the ith discrete time of τ. In addition, there is another commonly used metric describing frequency fluctuations which is the Allan variance $\sigma(\tau)$[22]:

$$\sigma(\tau) \cong \left[\frac{1}{2} \cdot \frac{1}{N-1} \sum_{i=1}^{N} \left(\frac{\overline{f_{i+1}} - \overline{f_i}}{f_0} \right)^2 \right]^{\frac{1}{2}}. \quad \text{(Eq. S24)}$$

We track the resonance frequency of the device using a closed-loop measurement setup for 300 seconds, with the results shown in Figure S19. We obtain from the data an RMS fractional frequency shift $\langle \delta f_0 / f_0 \rangle_\tau$ = 25.38 ppm (corresponding to an absolute frequency shift of 233.25 Hz) and an Allan variance $\sigma(\tau)$ = 10.09 ppm (corresponding to an absolute frequency shift of 92.73 Hz).

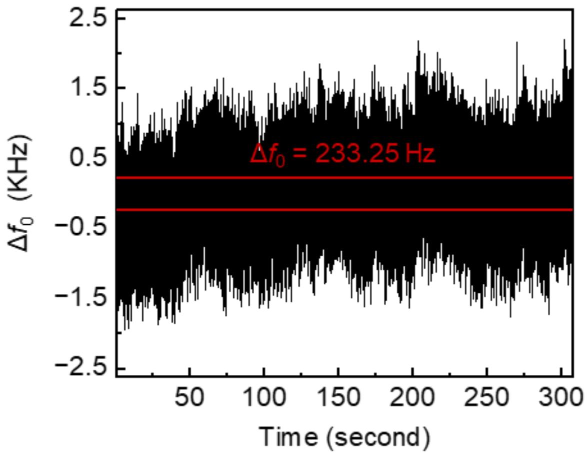

Figure S19. Measured frequency stability of the 2D β-In_2S_3 Resonator. The RMS fractional frequency shift under an integration time τ of 1 second is 25.38 ppm (red lines), which corresponds to an absolute frequency shift of 233.25 Hz.

Section S14. Reproducibility of the Resonance Measurements

We repeat the measurements multiple times to examine their reproducibility. Specifically, for the “position” measurements, we dynamically sweep the laser spot along the device diameter back and forth for 10 times (Figure S20(a)), and the measurement shows excellent reproducibility.

We further confirm that the use of “position” curve to extract device thermal properties is not affected by the choice of laser power (also suggested by theory). As shown in Figure S20(b), the “position” curve method yields similar results across different laser power values, demonstrating the robustness and reproducibility of our approach.

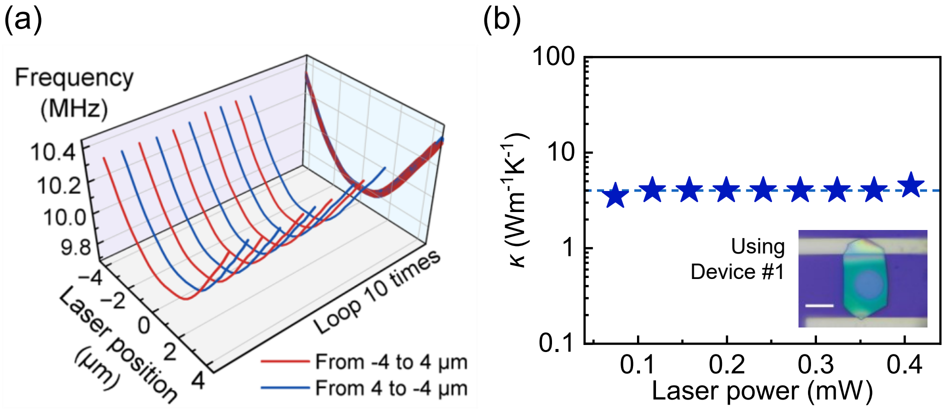

Figure S20. Demonstrations of reproducibility in frequency measurements and subsequent κ extraction. (a) Repeated measurements of sweeping laser position along a device diameter over 10 times. **(b)** Extracted value of κ using different laser powers. Scale bar: 10 μm.

References

1 Zhu J, Wang L, Wu J *et al.* Achieving 1.2 fm/Hz$^{1/2}$ displacement sensitivity with laser interferometry in two-dimensional nanomechanical resonators: Pathways towards quantum-noise-limited measurement at room temperature. *Chinese Phys Lett* 2023; **40**: 038102.

2 Blake P, Hill E W, Castro Neto A H *et al.* Making graphene visible. *Appl Phys Lett* 2007; **91**: 063124.

3 Wang Z, Lee J and Feng P X-L. Spatial mapping of multimode Brownian motions in high-frequency silicon carbide microdisk resonators. *Nat Commun* 2014; **5**: 5158.

4 El-Nahass M M, Khalifa B A, Soliman H S *et al*. Crystal structure and optical absorption investigations on β-In_2S_3 thin films. *Thin Solid Films* 2006; **515**: 1796–1801.

5 Malitson I H. Interspecimen comparison of the refractive index of fused silica. *J Opt Soc Am* 1965; **55**: 1205.

6 Palik E D. *Handbook of Optical Constants of Solids*. Cambridge: Academic Press, 1998.

7 Anders H. *Thin Films in Optics*. London: Focal Press, 1967.

8 Wah T. Vibration of circular plates. *J Acoust Soc Am* 1962; **34**: 275–281.

9 Lee J, Wang Z, He K *et al.* High frequency MoS_2 nanomechanical resonators. *ACS Nano* 2013; **7**: 6086–6091.

10 King GSD. The space group of β-In_2S_3. *Acta Cryst* 1962; **15**: 512–512.

11 Ding Y, Chen M, Gao X *et al*. Theoretical investigation on the electronic structure, elastic properties, and intrinsic hardness of Si_2N_2O. *Chinese Phys B* 2012; **21**: 067101.

12 Suzuki H, Yamaguchi N and Izumi H. Theoretical and experimental studies on the resonance frequencies of a stretched circular plate: Application to Japanese drum diaphragms. *Acoust Sci Technol* 2009; **30**: 348–354.

13 Zhu J, Xu B, Xiao F *et al.* Frequency scaling, elastic transition, and broad-range frequency tuning in WSe_2 nanomechanical resonators. *Nano Lett* 2022; **22**: 5107–5113.

14 Murakami Y. *Theory of Elasticity and Stress Concentration*. New York: John Wiley & Sons, 2016.

15 Islam A, van den Akker A and Feng P X-L. Anisotropic thermal conductivity of suspended black phosphorus probed by opto-thermomechanical resonance spectromicroscopy. *Nano Lett* 2018; **18:** 7683–7691.

16 Liu G and Zhou J. First-principles study of thermal expansion and thermomechanics of group-V monolayers: blue phosphorene, arsenene, and antimonene. *J Phys: Condens Matter* 2019; **31**: 065302.

17 Barton R A, Storch I R, Adiga V P *et al.* Photothermal self-oscillation and laser cooling of graphene optomechanical systems. *Nano Lett* 2012; **12**: 4681–4686.

18 Gokhale V J and Rais-Zadeh M. Uncooled infrared detectors using gallium nitride on silicon micromechanical resonators. *J Microelectromech Syst* 2014; **23**: 803–810.

19 Yang R, Wang Z and Feng P X-L. Calibrating temperature coefficient of frequency (TCf) and thermal expansion coefficient (α) of MoS_2 nanomechanical resonators. Joint Conference of the IEEE International Frequency Control Symposium & the European Frequency and Time Forum (IFCS 2015), Denver CO, USA, 12−16 April 2015.

20 Yang R, Wang Z and Feng P X-L. All-electrical readout of atomically-thin MoS_2 nanoelectromechanical resonators in the VHF band. 29th IEEE International Conference on Micro Electro Mechanical Systems (MEMS 2016), Shanghai, China, 24–28 January 2016.

21 Islam A, Lee J and Feng P X-L. Black phosphorus NEMS resonant infrared (IR) detector. 33rd IEEE International Conference on Micro Electro Mechanical Systems (MEMS 2020), Vancouver BC, Canada, 18–22 January 2020.

22 Feng P X-L. Ultra-high frequency nanoelectromechanical systems with low-noise technologies for single-molecule mass sensing. *Doctoral Thesis*. California Institute of Technology. 2006.
